# Rapidly self-deoxygenating controlled radical polymerization in water *via in situ* disproportionation of Cu(i)†

**DOI:** 10.1039/d0sc01512a

**Published:** 2020-05-05

**Authors:** Evelina Liarou, Yisong Han, Ana M. Sanchez, Marc Walker, David M. Haddleton

**Affiliations:** University of Warwick, Department of Chemistry Library Road Coventry CV4 7AL UK d.m.haddleton@warwick.ac.uk; University of Warwick, Department of Physics Coventry CV4 7AL UK

## Abstract

Rapidly *self-deoxygenating* Cu-RDRP in aqueous media is investigated. The disproportionation of Cu(i)/Me_6_Tren in water towards Cu(ii) and highly reactive Cu(0) leads to O_2_-free reaction environments within the first seconds of the reaction, even when the reaction takes place in the open-air. By leveraging this significantly fast O_2_-reducing activity of the disproportionation reaction, a range of well-defined water-soluble polymers with narrow dispersity are attained in a few minutes or less. This methodology provides the ability to prepare block copolymers *via* sequential monomer addition with little evidence for chain termination over the lifetime of the polymerization and allows for the synthesis of star-shaped polymers with the use of multi-functional initiators. The mechanism of *self-deoxygenation* is elucidated with the use of various characterization tools, and the species that participate in the rapid oxygen consumption is identified and discussed in detail.

## Abstract 2

The rapidly self-deoxygenating Cu-RDRP in aqueous media is investigated.

## Graphical abstract

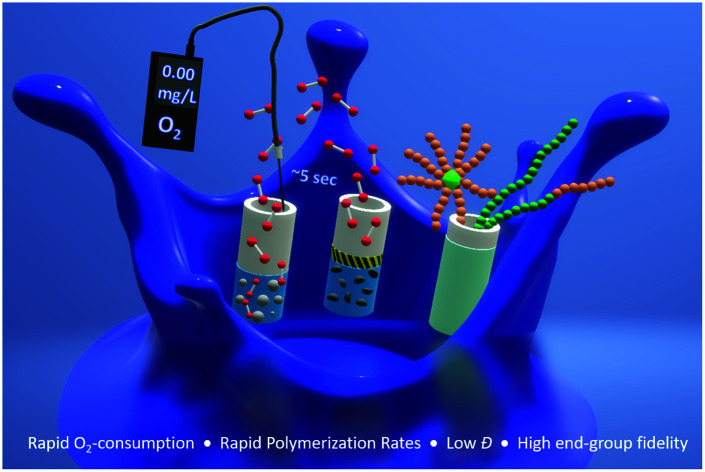

## Introduction

Reversible Deactivation Radical Polymerization (RDRP) methods, including atom-transfer radical polymerization (ATRP),^1,2^ single electron transfer-living radical polymerization (SET-LRP),^3,4^ reversible addition–fragmentation chain-transfer polymerization (RAFT)^5,6^ and nitroxide-mediated polymerization (NMP)^7–9^ have provided access to an increasing range of well-defined materials with sophisticated architectures, various functionalities and controlled (macro)molecular characteristics.^10–12^ Until recently, a notable hindrance for their development has been oxygen intolerance. Consequently, controlled radical polymerization processes conducted in the presence of oxygen are inhibited due to the ability of oxygen to react with carbon-centred radicals, leading to the formation of peroxy radicals *via* side reactions.^13,14^

Although the traditionally applied deoxygenation approaches, including freeze–pump–thaw cycles and N_2_/Ar sparging are unarguably efficient for O_2_ removal, they can be disadvantageous when volatile reagents are deoxygenated leading to their evaporation, and they are incompatible with new polymerization platforms (*i.e.* high-throughput) were the reaction scales are low.^15,16^ In this context, research has been focused on the replacement of these traditional methods either chemically through oxygen scavengers and reducing agents (*i.e.* glucose oxidase (GO_*x*_),^17–20^ ascorbic acid,^21–23^ hydrazine,^24,25^ photoredox catalysts^26,27^) or by physically displacing oxygen through headspace elimination.^28–31^

In particular, the reaction medium plays an important role in the evolution of an *oxygen tolerant* polymerization since the concentration of dissolved O_2_ varies in different solvents and can also exhibit differences depending on temperature, solution viscosity and pressure.^32–34^ Although oxygen tolerance and consumption have been thoroughly investigated in organic media,^14,24,25,28–31,35–38^*oxygen tolerant* polymerization in aqueous media can be considered challenging. Apart from the high concentration of dissolved O_2_ in even purified water (>8 mg L^−1^ at ambient temperature), there is high potential of side reactions, such as hydrolysis or elimination of the R–X or P–X bond, dissociation of the deactivating Cu(ii) species when Cu-RDRP is applied,^39,40^ and hydrolysis of the (macro-)chain transfer agent (CTA or macro-CTA)^41–43^ in the case of RAFT.^44,45^ Consequently, *oxygen tolerant* polymerizations in aqueous media require the efficient removal of oxygen from the polymerization solution, and also necessitate rapid rates in order to avoid these chain termination events.

In order to avoid conventional deoxygenation in aqueous-mediated RAFT photopolymerization of *N*,*N*-dimethylacrylamide, Boyer and colleagues reported the use of a zinc porphyrin photocatalyst (ZnTPPS^4−^) with ascorbic acid as a singlet oxygen quencher.^46^ In a separate study, the same group demonstrated the combination of eosin Y with ascorbic acid as a reducing agent system, for the oxygen tolerant aqueous RAFT photopolymerization at low volumes.^23^ In the field of Cu-mediated polymerization, among other elegant approaches,^47,48^ He and co-workers reported the addition of Cu(0) and ascorbic acid as reducing agents, for the surface-initiated ATRP of 2-hydroxyethyl methacrylate.^49,50^ Matyjaszewski and colleagues reported on the conversion of O_2_ to CO_2_ through GO_*x*_, for the aqueous ATRP of oligo(ethylene oxide) methyl ether methacrylate (OEOMA_500_) in the presence of pyruvates^51^ or horseradish peroxidase.^52^ Percec has reported the Cu(0) wire-mediated SET-LRP of methyl acrylate in the presence of air, with the addition of hydrazine as additional reducing agent.^24^ Recently, Bennetti and colleagues reported an oxygen tolerant Fe(0) system for the SI-ATRP of polymer brushes where the iron acts both as a source of catalyst and as a reducing agent showing excellent cytocompatibility towards mammalian cells for preparation of biomaterials.^53^ Theodorou *et al.* have reported a versatile, oxygen tolerant photoinduced Cu-RDRP approach in emulsion for the synthesis of various protein–polymer bioconjugates, with different proteins used as macroinitiators and plastic syringes as reaction vessels, in order to efficiently eliminate headspace. Their approach was compatible with various monomers, proteins and reaction conditions, achieving well-defined bioconjugates within 2 hours. ^54^

**Scheme 1:**
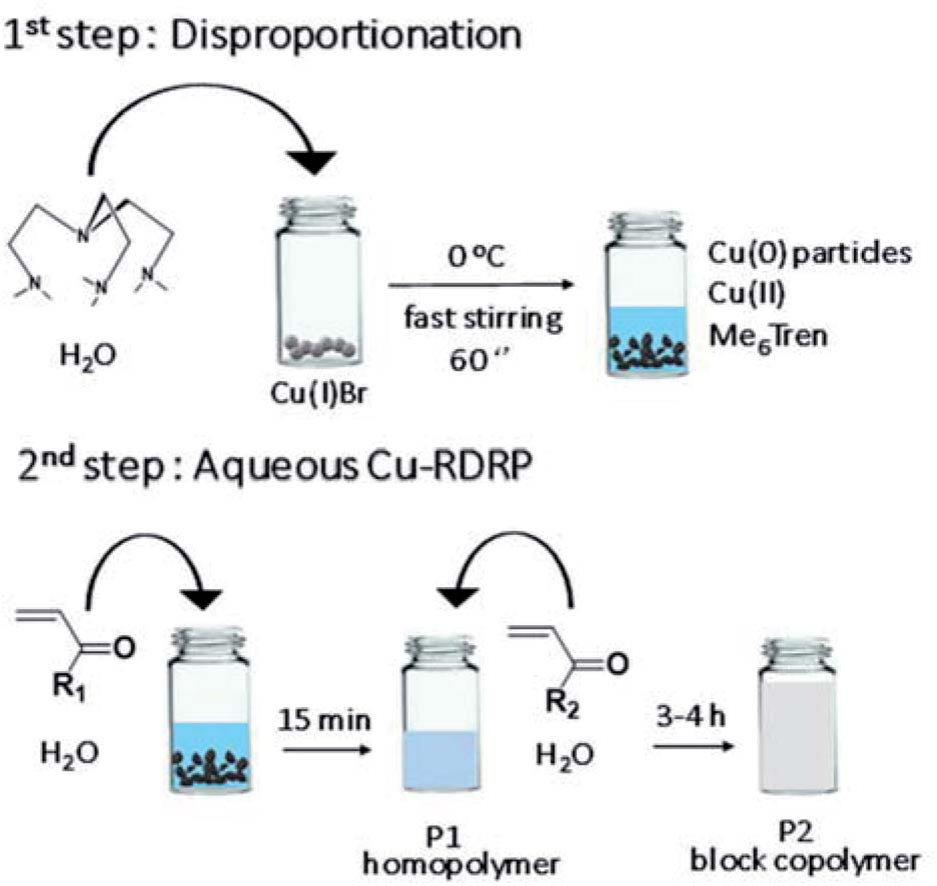
Schematic representation of the *self-deoxygenating* aqueous Cu-RDRP of acrylamides employing the pre-disproportionation of Cu(i)Br/Me_6_Tren.

Herein, we report the first instantaneously *self-deoxygenating* Cu-RDRP of various monomers in aqueous media, by omitting any type of conventional deoxygenation method (Scheme 1). The disproportionation of Cu(i) in the presence of Me_6_Tren as a tertiary amine aliphatic sigma-donor ligand in water towards Cu(0) and Cu(ii) is exploited, leading to full oxygen consumption within seconds, both in sealed and open-air conditions. Owing to the rapid O_2_ reducing activity of the disproportionation reaction, a range of hydrophilic homo- and block co-polymers with controlled molecular weight, low dispersity and sufficient end-group fidelity are synthesized within minutes. The aqueous oxygen consumption profile is elucidated by the *in situ* online monitoring of the dissolved [O_2_] through a fibre-optic oxygen monitoring probe, and the effect of the catalyst and ligand concentration, as well as the effect of different solvents are presented, and the mechanism of *self-deoxygenation* is discussed in detail. The findings are corroborated with a number of analytical methods which collectively verify the rapid oxidation of Cu-species and unravel the nature of the oxidized products.

## Results and discussion

**Fig. 1:**
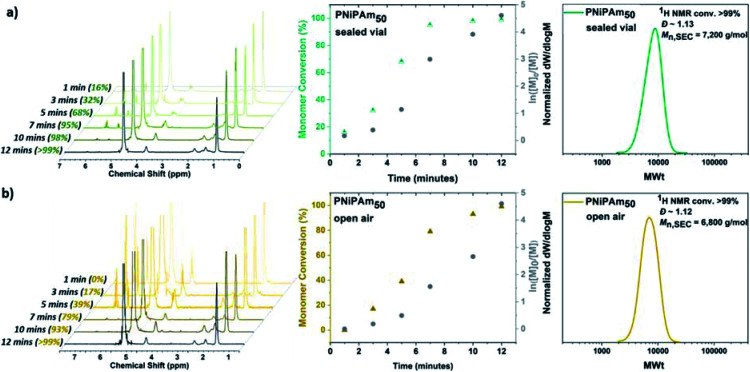
Kinetic studies for the self-deoxygenating aqueous Cu-RDRP of NiPAm with targeted DP_*n*_ = 50 when the polymerization was carried out (a) in sealed vial and (b) open-to-air conditions with ^1^H NMR spectra (left), plots of conversion and ln([M]_0_/[M]) over time (middle) and DMF-SEC derived molecular weight distributions (right).

Initially, in order to examine the ability to carry out efficient polymerization reactions in the presence of oxygen, the aqueous Cu-RDRP of *N*-isopropylacrylamide (NiPAm) was conducted without any type of external deoxygenation (Scheme S1†), using only headspace elimination, following the conditions [I] : [NiPAm] : [Cu(i)Br] : [Me_6_Tren] = 1 : 50 : 0.4 : 0.4. For this purpose, a sealed 8 mL glass vial was charged with Cu(i)Br, 1 mL H_2_O and Me_6_Tren, and was placed in ice-bath with rapid stirring (900 rpm) for 60 seconds. Upon formation of a heterogeneous blue solution (indicative of the formation [Cu(H_2_O)_6_]^2+^) with a black-purple Cu(0) precipitate, an aqueous solution of NiPAm and the water-soluble initiator (2,3-dihydroxypropyl 2-bromo-2-methylpropanoate) were added to the disproportionation solution, and the polymerization was left to commence. Kinetic studies were performed to reveal rapid polymerization rates, with >99% monomer conversion only after 12 minutes in accordance with previous results,^55^ as well as low dispersity being achieved (*Ð* = 1.15) and good agreement between experimental and theoretical *M*_*n*_ values verifying the versatility to carry out the polymerization without prior removal of dissolved oxygen (Fig. 1a). Furthermore, when the PNiPAm_50_ from this non-deoxygenated reaction was compared with the N_2_-sparged deoxygenation, good agreement was observed between both the two *M*_*n*,SEC_ values and the dispersities (Table 1, Fig. S1 and S2†).

**Table 1:** ^1^H NMR and DMF-SEC analysis for PNiPAm with targeted DP_*n*_ = 50 synthesized *via* N_2_-deoxygenated, non-deoxygenated (sealed vial) and open-to-air aqueous Cu-RDRP^a^

Conditions	Conv. b(%)	*M*_*n*,SEC_ c	*M*_*n*,th_ (g mol^−1^)	*Đ*	Reaction time
N_2_ sparging	>99	6200	5900	1.10	12 min
ND/sealed	>99	7200	5900	1.13	12 min
ND/open-air	>99	6800	5900	1.12	12 min

a In all polymerizations, the monomer concentration was 10 w/v % and the conditions were [I] : [DP_*n*_] : [Cu(i)Br] : [Me_6_Tren] = 1 : 50 : 0.4 : 0.4.

b Conversion was calculated *via*^1^H NMR in D_2_O.

c Determined by DMF-SEC analysis and expressed as molecular weight equivalents to PMMA narrow molecular weight standards.

Based on previous work on Cu(0)-wire mediated-RDRP in organic solvents where it was found that elimination of the headspace was important for successful polymerization, we were interested in investigating the effect of the [O_2_] concentration. The aqueous Cu-RDRP of NiPAm with targeted DP_*n*_ = 50 was tested under open to air conditions, performing both the disproportionation of Cu(i) and the polymerization reaction without sealing the vial, whilst applying a fast stirring rate (900 rpm).

Although the polymerization reached near-quantitative conversion (>99%) after 12 minutes (similarly to the sealed reaction) an induction period, ascribed to continuous O_2_ diffusion into the reaction was observed, which was further assisted by the rapid stirring, as discussed in the mechanistic section below. Importantly, the robustness of the system was further verified when higher molecular weights were targeted, applying the same conditions *i.e.* low monomer concentration of 10 w/v % and lower temperature without any external deoxygenation. It has previously been reported that when DP_*n*_ ≥ 80 was targeted, the ratio [Cu(i)Br] : [Me_6_Tren] = 1 : 1 resulted in inefficient deactivation, which was observed as high dispersity values.^56^ Therefore, for PNiPAm with targeted DP_*n*_ = 100, 200 and 400 twice as much Cu(i)Br as for DP_*n*_ = 50 was used, following the conditions [I] : [M] : [Cu(i)Br] : [Me_6_Tren] = 1 : DP_*n*_ : 0.8 : 0.4. It should be noted that, although the polymerization with targeted DP_*n*_ = 50 reached >99% conversion after only 12 minutes, for higher molar masses longer reaction times were required with DP_*n*_ = 200 taking 60 minutes to reach >99% conversion and DP_*n*_ = 400 taking up to 90 minutes (Table S1 and Fig. S3a†). Furthermore, when DP_*n*_ = 400 was initially targeted with 10 w/v % monomer concentration, the polymerization was not successful while when 20 w/v % (thus higher catalyst concentration) was used, high conversion, low dispersity and controlled molecular weights were obtained. In no case, was background, uncontrolled, polymerization observed, as verified by the SEC chromatograms from dual angle light scattering and viscosity detectors across the entire calibrated region (Fig. S3b†). The versatility of this methodology was expanded with the implementation of this aqueous *self-deoxygenating* system to both acrylates and acrylamides (Table 2), as well as more complex polymer architectures including star-shaped polymers.

**Table 2:** Macromolecular characteristics and reaction time of the various linear synthesized polymers^a^

Polymer	Conv. d(%)	*M*_*n*,SEC_ e	*M*_*n*,th_ g mol^−1^	*Đ*	Time (min)
PNiPAm_100_^b^	>99	16 200	11 600	1.14	15
PHEAm_100_^b^	>99	26 200	11 800	1.17	15
PDMA_80_^b^	>99	11 300	8200	1.15	20
P(PEGA_480_)_20_^c^	98	9300	9600	1.17	30
PNAM_40_^b^	98	7100	5800	1.09	240

a The monomer concentration = 10 w/v %.

b [Cu(i)Br] : [Me_6_Tren] = [0.8] : [0.4].

c [Cu(i)Br] : [Me_6_Tren] = [0.4] : [0.4].

d Conversion calculated *via*^1^H NMR in D_2_O.

e Determined by DMF-SEC analysis and expressed as molecular weight equivalents to PMMA narrow molecular weight standards.

NiPAm, *N*-hydroxyethyl acrylamide (HEAm) (Fig. S4†), *N*,*N* dimethylacrylamide (DMA) (Fig. S5†), poly(ethylene glycol) methyl ethyl acrylate (PEGA_480_) (Fig. S6†) and *N*-acryloylmorpholine (NAM) (Fig. S7†) were polymerized through aqueous Cu-RDRP, without applying any type of external deoxygenation, resulting in excellent control over the obtained *M*_*n*,SEC_ values, with low dispersity and near-quantitative conversions over short reaction times (Table 2). Additionally, since star-shaped polymers have gained a lot of interest due to their diverse properties,^10,57–59^ the *self-deoxygenating* approach was applied for the synthesis of star-shaped PHEAm polymers with overall targeted DP_*n*_ = 60, 80 and 120.

For this purpose, 3-, 4- and 8-arm multifunctional initiators were used for the synthesis of PHEAm star-shaped polymers with each arm having a targeted DP_*n*_ = 20. As the multi-arm star initiators were relatively hydrophobic, polymerizations were conducted in water – organic solvent mixtures (either methanol or DMSO), with the pre-disproportionation of Cu(i) being carried out in pure water in each case.^60^ As a result, PHEAm star-shaped polymers were obtained at near-quantitative conversions (>99%) and narrow molecular weight distributions (*Ð* = 1.11–1.2) in less than 2 hours (Fig. S8†). It should be noted that the higher than the theoretical *M*_*n*,SEC_ values for both linear and star-shaped PHEAm are attributed to interactions of the two –OH groups with the SEC-column and such deviations are observed for all the HEAm-derived polymers.

**Fig. 2:**
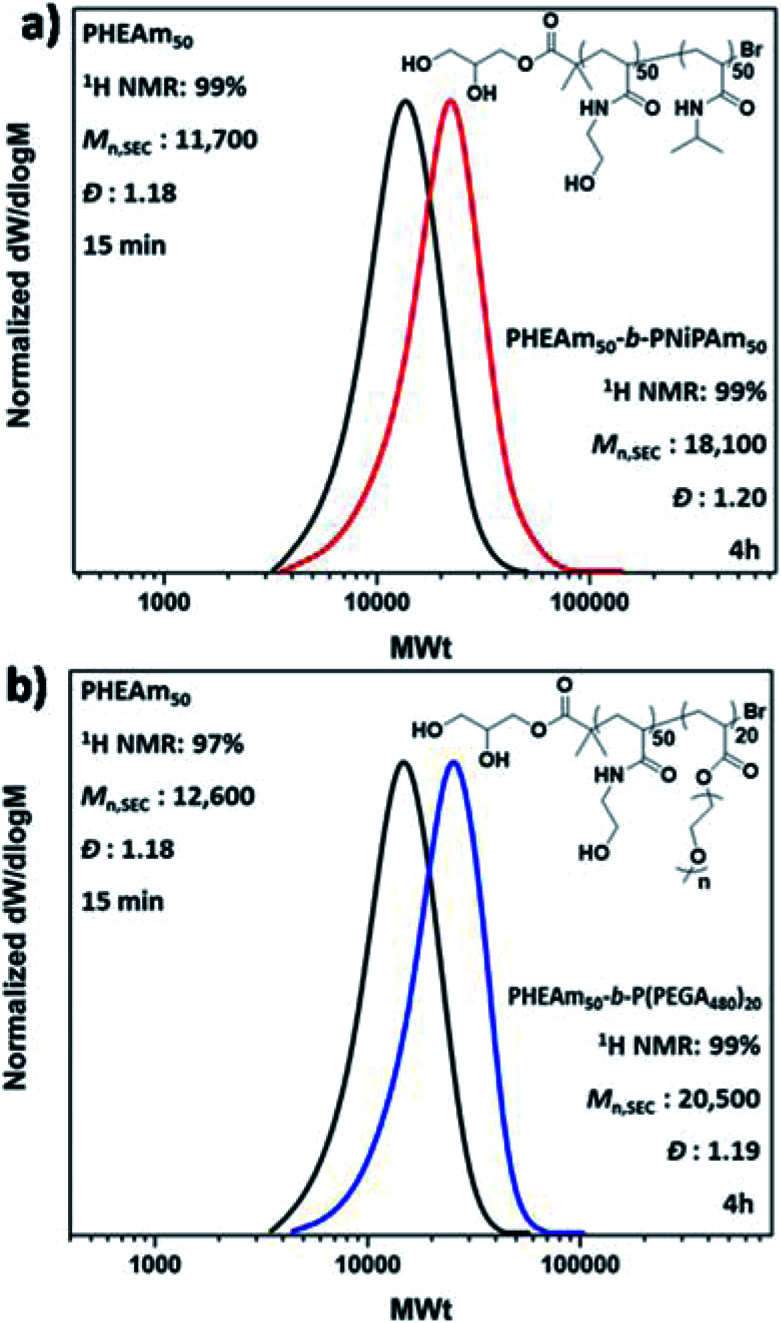
DMF-SEC derived molecular weight distributions for the *in situ* sequential monomer addition of the diblock copolymers (a) PHEAm_50_-*b*-PNiPAm_50_ and (b) PHEAm_50_-*b*-P(PEGA_480_)_20_.

Given the non-deoxygenated environment, as well as the side reactions which are well-known to occur in aqueous media, we were interested in investigating the extent of end group fidelity in this approach. For this purpose, MALDI-ToF-MS was employed for the mass characterization of PNiPAm with targeted DP_*n*_ = 50, synthesized in a sealed vial. The MALDI-ToF analysis revealed that there are peak distributions corresponding to polymer chains that had undergone both elimination and hydrolysis of the alkyl halide, whilst bromine capped chains were also observed (Fig. S9†). These chain termination events are also observed under conventionally deoxygenated systems^56,61,62^ originating from the excess of water where the rate of hydrolysis is lower than the rate of chain propagation, thus they cannot be correlated only with the presence or absence of oxygen. However, the analysis of the ^1^H NMR spectra of both PNiPAm which was analyzed through MALDI and the extreme case of PNiPAm synthesized in open air, showed no peaks corresponding to vinyl protons originating from elimination (Fig. S9b†). This observation led us to hypothesize that elimination might occur either during the MALDI process or during the sample preparation, and possibly is not occurring in the timescale of the polymerization. This hypothesis was further verified by performing *in situ* block copolymerizations which resulted in polyacrylamide diblock copolymers with well-defined molecular characteristics at high conversions (Fig. 2a and b). Although this rapid polymerization without loss of end group fidelity *via* radical–radical termination is not in accordance with classical free radical polymerization kinetics, Ballard and Asua have shown that when the probability density functions of the termination reactions are altered to allow for radical diffusion, the reduced rate of termination can explain “the ability for a seemingly impossible level of control of radical reactions”.^63^ The versatility of the methodology was further verified through low volume reactions carried out in 96-well plates which were sealed with a plate-lid. Both PNiPAm and HEAm were polymerized in 300 μL total reaction volume, exhibiting good control over the molecular weights and the dispersity at high conversion (Fig. S10†).

### Rate of oxygen consumption during Cu(i) disproportionation to Cu(ii) and Cu(0)

**Fig. 3:**
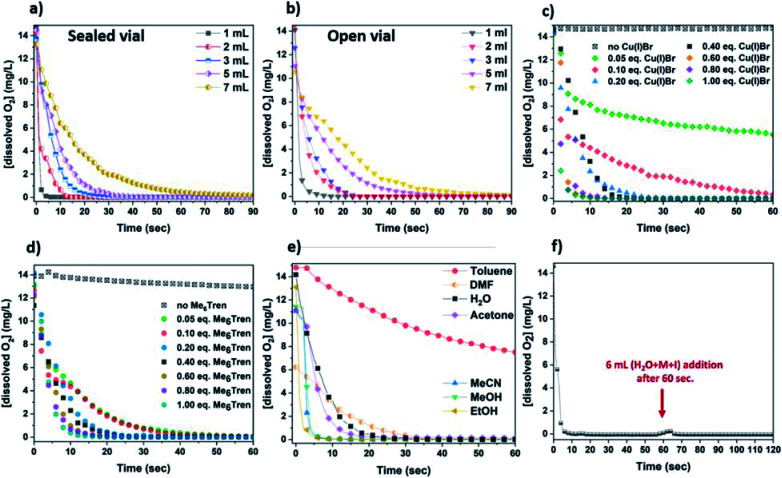
Line graphs illustrating the oxygen consumption over time with (a) different concentrations of the Cu(i)Br/Me_6_Tren complex in a sealed environment (with [Cu(i)Br] : [Me_6_Tren] = 1 : 1) and (b) open-to-air conditions (with [Cu(i)Br] : [Me_6_Tren] = 1 : 1), (c) various concentrations of Cu(i)Br in 3 mL reaction volume, (d) various concentrations of Me_6_Tren in 3 mL reaction volume, (e) in different solvents with 3 mL reaction volume and (f) upon addition of 6 mL aqueous solution of monomer and initiator in 1 mL disproportionation reaction. All measurements were conducted in glass vials with 7 mL capacity, at ∼0 °C and 900 rpm stirring rate.

The rate of the oxygen reducing activity of the Cu(i)/Me_6_Tren aqueous solution was investigated *via* the online monitoring of the dissolved O_2_ concentration with the use of an oxygen probe. Initially, the [dissolved O_2_] in the disproportionation solution used for the polymerization of NiPAm with targeted DP_*n*_ = 50 (with [Cu(i)] : [Me_6_Tren] = 1 : 1) was monitored, resulting in rapid (a few seconds) oxygen consumption (Fig. 3a – grey). Subsequently, since the concentration of the components able to consume oxygen affect the rate of oxygen consumption, different concentrations were examined, using 1–7 mL of H_2_O (Fig. 3a).

As expected, the rate of oxygen consumption exhibited differences depending on the concentration, with the fastest oxygen consumption (∼3 s) observed for the most concentrated solution (1 mL of H_2_O), and the slowest rate observed for 7 mL of H_2_O (∼1 min). It is noted that when 1 mL of the solution was used prior to monomer addition (HEAm in this case) and initiator addition, the polymerization exhibited the highest control over the molecular weights (Fig. S11 and Table S2†). This suggests that the fate of an aqueous non-deoxygenated polymerization is dependent on the concentration of the disproportionation reagents (Cu(i)Br/Me_6_Tren) and/or their products (Cu(0), Cu(ii)), and sufficient amount of these species is important in order to facilitate the O_2_ consumption process. In order to provide an insight into the open-to-air polymerization where almost perfect control over the macromolecular characteristics was observed, the [O_2_] monitoring was performed in an open vial.

In this case, the oxygen consumption was again rapid (∼10–60 s) (Fig. 3b), with the 1 mL solution becoming *self-deoxygenated* within the first 10 seconds. The trend of consumption exhibited the same profile as in the sealed vial, again depending on the concentration of the disproportionation solution. The main difference between the sealed and the open-to-air experiments was that the latter required slightly longer reaction times for total deoxygenation to occur, which could be attributed to the constant diffusion of O_2_ into the solution from the air during high stirring rates (Fig. 3a and b). It should be noted that at ∼0 °C (temperature applied at the disproportionation and polymerization reactions), the solubility of oxygen is higher than at ambient (*i.e.* 25 °C) temperature (∼14 mg L^−1^ and ∼8 mg L^−1^ respectively), while the diffusion rate of oxygen is lower.^64–66^ Hence, although there is a constant exposure of the reaction solution to air/oxygen, the reducing ability of Cu(0) (as well as Cu(i)), combined with the low-temperature conditions could further facilitate the *oxygen tolerant* nature of this aqueous Cu-RDRP.

Subsequently, the role of the ligand was investigated by using different amounts of Me_6_Tren with respect to Cu(i)Br. Initially, a solution containing only Cu(i)Br and 3 mL H_2_O was examined which, although being heterogeneous (due to the insolubility of Cu(i)Br in water), a very slow O_2_ consumption was observed (from 14 mg L^−1^ to ∼13 mg L^−1^ after 1 min). However, with the addition of low loadings of Me_6_Tren ([Cu(i)Br] : [Me_6_Tren] = 1 : 0.125) full O_2_ consumption was observed after ∼40 s (Fig. 3d – green and pink). Following this, higher concentrations of Me_6_Tren resulted in accelerated consumption rates (Fig. 3d), with the fastest O_2_ consumption being observed when excess of Me_6_Tren was applied ([Cu(i)Br] : [Me_6_Tren] = 1 : 2.5, Fig. 3d – dark cyan). This indicates that, upon formation of the Cu-complex with the amine ligand in water, the rapid disproportionation of Cu(i) is followed by rapid O_2_ consumption, even when low loadings of Cu(i)Br or ligand are used. In this context, it is speculated that even small amounts of the *in situ* generated Cu(0) particles can “consume” oxygen. However, since the “nascent” Cu(0) particles are highly reactive, we hypothesized that their generation is followed by their oxidation in the presence of O_2_ (*vide infra* electron microscopy and XPS).

In order to examine which of the Cu-species following disproportionation participate in the O_2_ consumption, the [O_2_] evolution was also monitored in different solvents including ethanol, methanol and dimethylformamide (DMF) which promote disproportionation of Cu(i),^67^ as well as solvents that stabilize Cu(i)^68^ and thus, disproportionation is not favoured (*i.e.* acetone, acetonitrile, toluene) (Fig. 3e). Specifically, when EtOH and MeOH that facilitate the disproportionation of Cu(i) were used, the fastest O_2_-consumption rates were observed. Noteworthy is that the same trend was observed when MeCN, which favours the stabilization of Cu(i), was used. Slower consumption was evidenced when acetone, H_2_O and the polar aprotic solvent DMF were used, while toluene where Cu(i) is insoluble, exhibited the slowest rates. Since acetonitrile stabilises Cu(i) and does not promote disproportionation, we speculate that O_2_ consumption occurs from the oxidation of Cu(i) to Cu(ii). Based on this, it is possible that O_2_ consumption is assisted by the oxidation of both Cu(i) and the “nascent” Cu(0), and depending on the choice of solvent, the oxidation of Cu(0) and Cu(i) are competing reactions which can both lead to rapid deoxygenation. The continuous deoxygenation profile (even after the addition of monomer and initiator solutions the [O_2_] has been reduced, and the O_2_-free environment is re-established) (Fig. 3f) might be attributed to the regeneration of Cu(i) upon oxidation of Cu(0), which can *re*-disproportionate and thus “perpetuate” the reduction of O_2_ until the reagents are fully consumed. As a result, it is hypothesized that Cu(i), originating either from the initially added Cu(i)Br or from the oxidation of Cu(0), and “nascent” Cu(0) participate in the consumption of both dissolved and in gas phase O_2_, and due to the long-lasting paucity of these Cu-species, the reaction solutions remain deoxygenated even open-to-air.

**Fig. 4:**
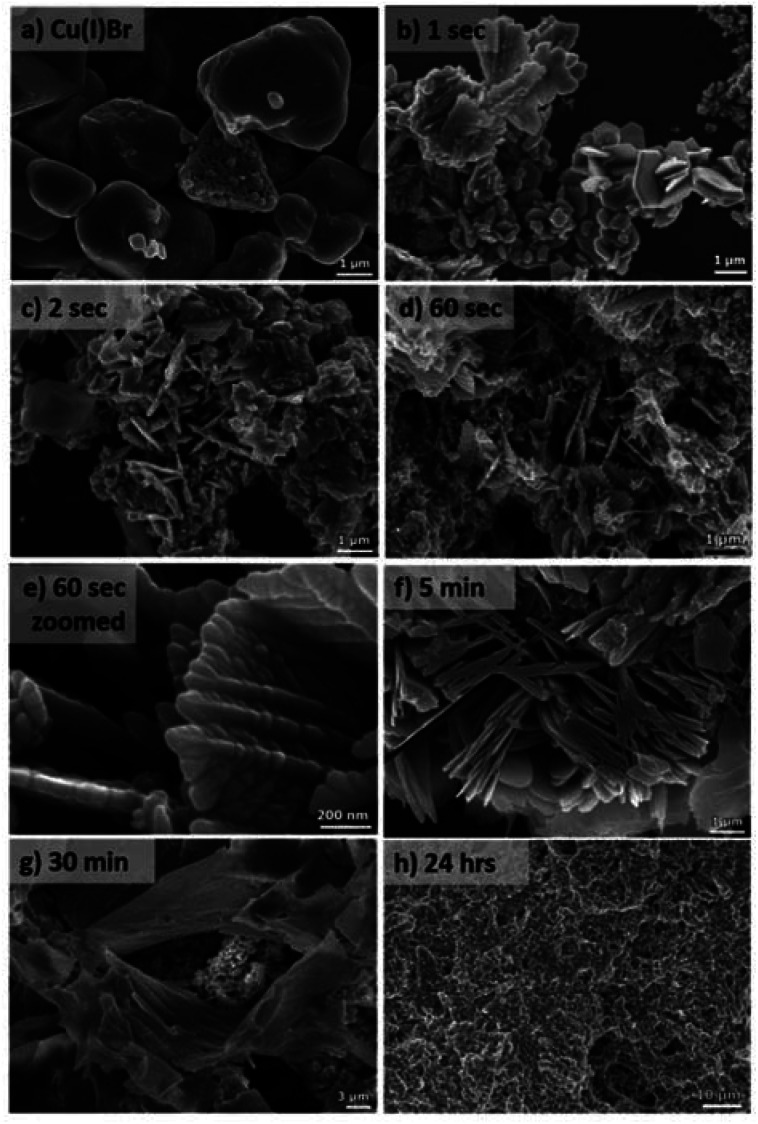
SEM images of (a) Cu(i)Br dispersed in H_2_O and (b–h) the *self-deoxygenating* disproportionation reaction precipitate collected at different times. The “∼1 s” was taken immediately after the addition of Me_6_Tren.

To better understand the effect of O_2_ on the catalyst and in an attempt to identify the oxidation products, XPS and EM (TEM, SEM, ADF-STEM and EELS) were employed. Initially, the solution following disproportionation in the presence of O_2_ was examined through SEM and aiming to monitor any changes in the morphology of the Cu particles over time, Cu(i)Br in water and aliquots of the disproportionation reaction were imaged (Fig. 4a–h). The Cu(i)Br sample in water consists of >1 μm size well-shaped aggregates. Upon addition of Me_6_Tren and after the ∼1^st^ second of the reaction, where there is an instantaneous observation of black/purple Cu(0) precipitate with fast stirring, the sample mainly includes three different structures, namely dendrite-like shaped aggregates which consist of particles >100 nm, small multi-sized particles, as well as faceted crystal structures (Fig. 4b). It is notable that the crystal-shaped morphologies are mainly present for the sample taken immediately after the addition of Me_6_Tren (“∼1 s” sample).

Considering the loss of the larger Cu(i)Br aggregates, it is hypothesized that the crystals are formed upon instant consumption of Cu(i)Br, following a clusterization process (Fig. S12 and S13†) which can be correlated with the formation of the dendrite-like shaped aggregates. After ∼2 seconds of the reaction, the observed faceted structures are significantly less, whilst the dendritic-like aggregates, which we hypothesize that follow a nucleation and growth process become more evident (Fig. 4c–e). It is notable that this morphology is still observed after 60 seconds of the reaction, while after 5 minutes this dendritic pattern becomes “softer”, with the dendritic-like branches becoming less evident and more uniform (Fig. 4f). Finally, after 30 minutes, small particle-like aggregates are observed which are covered by more uniform larger aggregates (Fig. 4g) and finally, after 24 hours of the reaction, large aggregates that consist of smaller particles are observed (Fig. 4h).

**Fig. 5:**
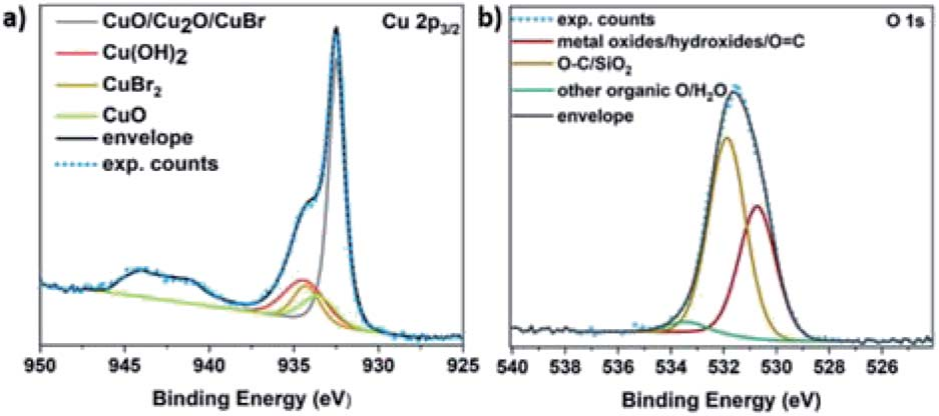
XPS core level (a) Cu 2p_3/2_ and (b) O 1s spectra of the disproportionation precipitate after 60 seconds of the reaction. The features between 940 eV and 945 eV (a) are due to shake-up peaks from Cu^2+^ states.

It is hypothesized that the consumption of O_2_ would lead to the possible formation of different copper oxides and thus, the EDX studies were focused on the presence of oxygen with respect to copper. Initially, Cu(i)Br was measured as blank sample, showing a distinctive peak at 0.9 keV corresponding to Cu, as well as a very small peak assigned to oxygen at 0.5 keV (Fig. S14†) and could be attributed to the oxidation of the Cu(i)Br powder from air over time. When the precipitate from the disproportionation was examined immediately after the addition of Me_6_Tren (sample was taken after ∼1 second), the peak at 0.5 keV, assigned to oxygen increased slightly (as well as after ∼2–3 seconds) and became even more evident after 60 seconds of the reaction (Fig. S14†). This observation might be related to the formation of oxides as the reaction takes place in the presence of oxygen, a hypothesis that is corroborated by the full O_2_ consumption within the first 60 seconds. After 30 minutes, the intensity of the oxygen peak further increased, with the highest intensity being observed after 24 hours. The presence of copper oxides and hydroxide was also verified through X-ray photoelectron spectroscopy (XPS) for both the black precipitate and the supernatant after 60 s. Examination of the Cu 2p_3/2_ region revealed the presence of Cu(ii) states in the precipitate (Fig. 5a), as evidenced by the shake-up features observed between 940 eV and 945 eV. Detailed fitting of this region and the peak around 934 eV using the work of Biesinger,^69^ showed evidence for Cu(OH)_2_, CuO and Cu(ii)Br_2_. Turning to the peak at 932.3 eV, it is not possible to distinguish between Cu(0) and Cu(i) states so one must look at alternative regions in order to understand the chemistry of the system. Fig. S15† presents the data from the Br 3d region, where two doublets were required to fit the data corresponding to Cu(i)Br and Cu(ii)Br_2_.

Next, the Cu LMM Auger emission region was analysed (Fig. S16†) and required the addition of the Cu(0) components as described by Biesinger to replicate the data.^69^ Comparing the Auger spectra of the precipitate and the supernatant, we observe a downward shift of around 2 eV in the kinetic energy of the peak intensity when moving from the precipitate to the supernatant, suggesting a higher Cu(ii) concentration in the supernatant. This hypothesis is corroborated by the Cu 2p_3/2_ spectrum acquired from the supernatant (Fig. S17a†), where the intensity of the shake-up features and the peak at 934.2 eV have both increased relative to the precipitate (Fig. 5a).

The O 1s data acquired also suggest the existence of both Cu(OH)_2_ and Cu oxides in both the supernatant (Fig. S17b†) and the precipitate (Fig. 5b). Overall, based on the elemental analysis of both the precipitate and supernatant (Tables S3 and S4†), copper and oxygen are mainly predominant in the precipitate, while nitrogen originating from the ligand (Fig. S18†), as well as bromine (originating from Cu(i)Br) are mainly present in the supernatant (Fig. S15†).

**Fig. 6:**
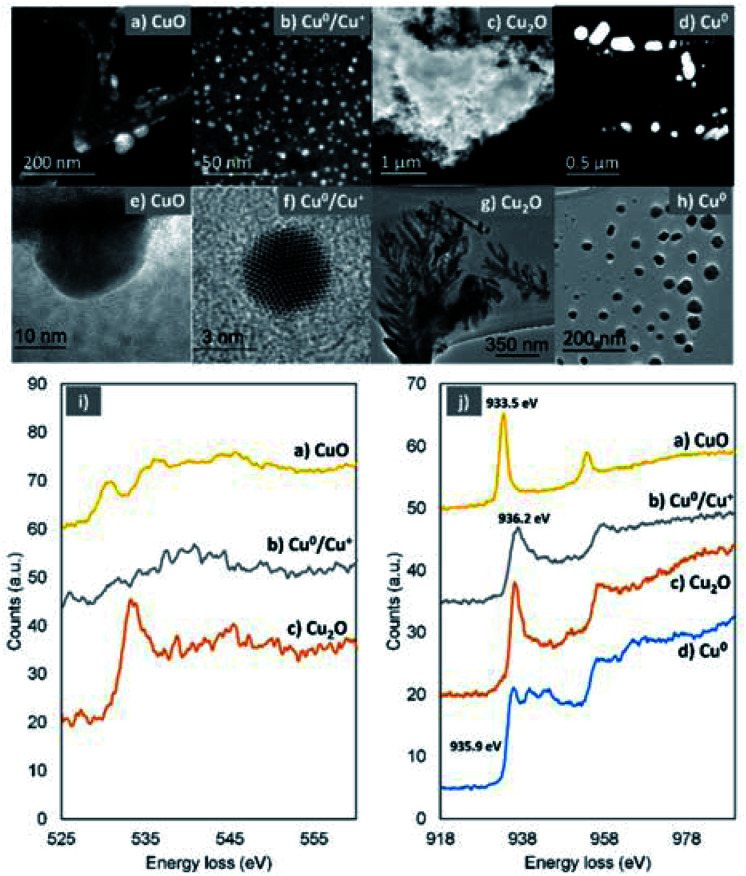
ADF-STEM images and the corresponding EELS spectra taken from several regions of the specimen showing the existence of different Cu-species after 60 seconds of the reaction. ADF-STEM images (a–d, top), TEM images (e–h, middle) and EELS spectra of O–K edge (i, bottom) from (a), (b) and (c), respectively, where no O signals were detectable from (d). EELS spectra of Cu–L edge (j, bottom) from (a), (b), (c) and (d), respectively. In view of the fine structures exhibited in the EELS spectra, it is confirmed that (a) is CuO, (c) is Cu_2_O and (d) is Cu(0). In (d), the Cu/O atomic ratio is estimated to be <2, indicating a mixture of Cu(0) and Cu_2_O.

High-resolution transmission electron microscopy (HR-TEM) and annular dark field-scanning transmission electron microscopy (ADF-STEM) in combination with electron energy loss spectroscopy (EELS) were used in order to provide more details on the chemical state of the Cu-species generated upon reaction with oxygen. The HR-TEM of the disproportionation precipitate (60 seconds aliquot) verified the presence of copper species with dramatically different morphology in the nanoscale, *i.e.* dendrite-like shaped structures (Fig. 6c and g), big particles with well-defined facets (Fig. 6d and h) and much smaller particles (Fig. 6a, b, e and f).

Based on the EELS analysis, the faceted particles exhibited a Cu–L_3_ edge peaked at 935.9 eV (Fig. 6j, blue) but no detectable O–K edge. This suggests that these particles are metallic Cu, which has remained unaffected from the presence of oxygen within the first 60 seconds of the reaction. In contrast, the dendrite-like structures exhibited a prominent O–K edge peaked at 533.8 eV and a Cu–L_3_ edge peaked at 936.2 eV, indicating the formation of Cu(i) materials (Fig. 6j, red).^70,71^

It is therefore speculated that these dendritic structures exist in the form of Cu_2_O which, based on their immediate presence upon addition of the ligand (after ∼1 second), were rapidly formed. The Cu–L_3_ edge from the smaller (<80 nm) particles is shifted to a lower energy loss of 933.5 eV (Fig. 6j, yellow). In combination with the energy loss features shown in the O–K edge (Fig. 6j, yellow), it is confirmed that these particles are CuO.^70,71^ In addition, even smaller (2–10 nm) particles were found (Fig. 6b and f) within the samples. They show a Cu–L_3_ edge corresponding to Cu(i) and a not clearly defined O–K edge. However, the analysis of the relative intensities between the O and Cu edges revealed that there is shortage of O within the very small particles and they are in fact a mixture of Cu(i) and Cu(0).

In summary, the fast oxygen consumption rates observed from the online O_2_-monitoring suggest that the solution following disproportionation can be considered as a rapidly self-deoxygenating system. In order to investigate the effect of the ligand and the Cu-source (Cu(i)Br) on oxygen consumption, different concentrations of these components were examined individually. In the absence of Cu(i)Br (when only Me_6_Tren is present) no oxygen consumption was observed over the timescale of the disproportionation reaction, while a gradual increase of the [Cu(i)Br] led to progressively faster rate of O_2_-consumption. In the absence of ligand (when only Cu(i)Br was present), a slightly descending trend was observed which became evident with the addition of even low loadings of Me_6_Tren. It is noteworthy that although the different concentrations of Cu(i)Br have a gradual effect on the consumption rate, alterations on the ligand concentrations did not show the same effect. This observation leads us to hypothesize that the rate of O_2_-consumption is mainly dependent on the concentration of Cu(i) which is directly related to the concentration of *in situ* generated highly reactive Cu(0) particles. Although the ligand cannot be considered as the primary O_2_-reducing agent based on these findings, its role is highlighted when negligible oxygen levels are observed even after the addition of monomer and initiator. This might be attributed to the ability of the ligand either to reduce Cu(ii) back to Cu(i), or *re*-initiate a new “disproportionation” cycle, thus leading to elimination of dissolved O_2_. To further clarify the role of the disproportionation components, the O_2_-consumption profile of the reaction was investigated in various solvents. It was shown that fast O_2_-consumption rates were observed not only for solvents that favour disproportionation of Cu(i) into Cu(0) and Cu(ii), but also for solvents that stabilize Cu(i) *i.e.* acetonitrile. These data indicate that both Cu(i) and Cu(0) can consume oxygen *via* being oxidised, and the oxidation of Cu(0) and Cu(i) are competing reactions which both lead to rapid deoxygenation.

These observations were further corroborated when electron microscopy (SEM, HR-TEM) and spectroscopic (XPS, EDX, EELS) techniques were collectively employed for the determination of the oxidized products. SEM along with EDX analyses indicated that within the first seconds of the reaction there is formation of copper oxides which becomes more evident as the reaction progresses, supporting our hypothesis that the copper species are primarily responsible for oxygen consumption. The morphological differences of the copper species facilitated further analysis through HR-TEM and EELS spectroscopy, which identified the different copper species. Characterization of both the reaction supernatant and precipitate through XPS revealed again that copper oxides are the main products of this self-deoxygenation process.

## Conclusions

The rapidly *self-deoxygenating* Cu-RDRP in aqueous media of various linear and non-linear, homo- and block-copolymers is reported. Disproportionation of Cu(i)/Me_6_Tren in water towards Cu(ii) and highly reactive Cu(0), leads to O_2_-free reaction environments within the first seconds of the reaction, even when the reaction is taking place in the open-air. By leveraging this significantly fast O_2_-reducing activity of the disproportionation reaction, well-defined water-soluble polymers with very narrow dispersity are attained in a few minutes or less. Importantly, this methodology provides the ability to prepare block copolymers *via* sequential monomer addition with little evidence for chain termination over the lifetime of the polymerization and allows for the synthesis of star-shaped polymers with the use of multi-functional initiators. The use of a range of characterization tools provides insights into this *self-deoxygenating* platform and identifies the species that participate in the oxygen consumption, as well as the species generated upon exposure of the solution to O_2_-rich environments. The unprecedentedly fast reducing ability of the Cu(i) disproportionation can be an advantageous and mild platform for applications that require instantaneous O_2_-free environments for long periods.

## Conflicts of interest

There are no conflicts to declare.

## Supplementary Material

SC-011-D0SC01512A-s001
