# Supplementary material for: Rapidly self-deoxygenating controlled radical polymerization in water via in situ disproportionation of Cu(i)
Source: Chem Sci. 2020 May 5;11(20):5257–66. doi: 10.1039/d0sc01512a (PMC8159280; doi:10.1039/d0sc01512a)
Supplement: SC-011-D0SC01512A-s001 [file SC-011-D0SC01512A-s001.pdf]

Electronic Supplementary Material (ESI) for Chemical Science.
This journal is © The Royal Society of Chemistry 2020

Supporting Information

Rapidly Self-deoxygenating Controlled Radical Polymerization in water *via in-situ* Disproportionation of Cu(I)

Evelina Liarou,[a] Yisong Han,[b] Ana Sanchez,[b] Marc Walker [b] and *David M. Haddleton[a]**

[a]University of Warwick, Department of Chemistry, Library Road, Coventry, CV4 7AL, UK

[b] Department of Physics, University of Warwick, Coventry, CV4 7AL, UK

email: d.m.haddleton@warwick.ac.uk

Experimental

Materials

The monomers *N*-isopropyl acrylamide (NiPAm, ≥ 99%), *N*-hydroxyethyl acrylamide (HEAm, 97%), *N, N* dimethylacrylamide (DMA, 99%), poly(ethylene glycol) methyl ethyl acrylate ($PEGA_{480}$, 97%), *N*-acryloylmorpholine (NAM, 97%) were used as received and without removal of the inhibitor. Solvents were purchased from Sigma Aldrich / Merck and used as received. For all the disproportionation reactions and the polymerization reactions distilled water was used. *Tris*(2-(dimethylamino)ethyl)amine (Me_6Tren) was synthesized according to the literature[1] and stored under N_2 atmosphere prior to use. The water-soluble initiator 2, 3-dihydroxypropyl 2-bromo-2-methylpropanoate was synthesized according to the literature.[2] The 3-arm,[3] 4-arm[4] and 8-arm[3] initiators were synthesized according to the literature. Copper (I) bromide (CuBr, 98%, Sigma-Aldrich) was washed with acetic acid and ethanol, dried under vacuum, and stored under N_2 atmosphere.

Instrumentation & Techniques

^{1}H Nuclear Magnetic Resonance.

^{1}H NMR spectra were recorded on Bruker DPX-300 or DPX400 spectrometers in deuterated dimethyl sulfoxide (DMSO-d_6) or deuterium oxide (D_2O) obtained from Sigma-Aldrich. Chemical shifts are given in ppm downfield from the internal standard tetramethylsilane. Monomer conversions were determined via ^{1}H NMR spectroscopy by comparing the integrals of monomeric vinyl protons to polymer signals.

Size Exclusion Chromatography

<u>DMF</u>

Agilent Infinity II MDS instrument equipped with differential refractive index (DRI), viscometry (VS), dual angle light scatter (LS) and variable wavelength UV detectors. The system was

equipped with 2 x PLgel Mixed D columns (300 x 7.5 mm) and a PLgel 5 µm guard column. The eluent used was DMF with 5 mmol NH_4BF_4 additive to reduce column interactions. Samples were run at 1 ml/min at 50 ^oC. Poly(methyl methacrylate) standards (Agilent EasyVials) were used for calibration between 955,000 – 550 g mol^{-1}. Analyte samples were filtered through a nylon membrane with 0.22 µm pore size before injection. Respectively, experimental molar mass ($M_{n,SEC}$) and dispersity ($Đ$) values of synthesized polymers were determined by conventional calibration and universal calibration using Agilent GPC/SEC software.

Matrix-assisted laser desorption/ionization time-of-flight

(MALDI-ToF-MS) measurements were conducted using a Bruker Daltonics Ultraflex II MALDI-ToF mass spectrometer, equipped with a nitrogen laser delivering 2 ns laser pulses at 337 nm with positive ion ToF detection performed using an accelerating voltage of 25 kV. Aliquots (50 µL) from the reactions were diluted in tetrahydrofuran (THF) (50 µL) of trans-2-[3-(4-tert-butylphenyl)-2-methyl-2-propyldene] malononitrile (DCTB) or α-cyano-4-hydroxycinnamic acid (CHCA) as a matrix (saturated solution), sodium iodide as the cationization agent (1.0 mg mL^{-1}) and sample (1.0 mg mL^{-1}) were mixed, and 0.7 µL of the mixture was applied to the target plate. Spectra were recorded in reflectron mode calibrated with poly(ethylene glycol) monomethyl ether (PEG-Me) 1,900 kDa.

Scanning Electron Microscopy (SEM) and energy-dispersive X-ray (EDX) spectroscopy

Scanning electron microscopy was performed using a ZEISS Gemini SEM - Field Emission Scanning Electron Microscope and a ZEISS Supra. Best results were obtained when using the InLens detector with ~3.5 mm working distance, 20 (Gemini) or 30 (Supra) µm aperture and 5-15 kV acceleration voltage, with respect to sample tolerance. EDX spectroscopy and elemental analysis were performed *via* the Gemini instrument through its SDD EDX detector.

Sample Preparation: A 3 mL total capacity glass vial placed in an ice bath was charged with 7.2 mg (1 eq.) Cu(I)Br and 1 mL DI-H_2O and was septum-sealed. Upon fast stirring (900 rpm),

Me_6Tren (14 µL, 1 eq.) was added in the Cu(I)Br solution and aliquots from the heterogeneous solution were dropcast on silicon wafer chips (5 mm x 7 mm) which were attached to aluminium specimen stubs. The dropcast samples were instantly being placed under N_2 blanket and left to dry.

Transmission Electron Microscopy (TEM) imaging and Electron Energy Loss Spectroscopy (EELS)

TEM imaging was carried out using a JEOL 2100 electron microscope. Annular dark-field STEM imaging and EELS spectrum imaging were performed in a double-corrected JEOL ARM200f microscope, equipped with a Gatan Quantum spectrometer, operated at 200 kV. A probe convergence semi-angle of 32 mrad and a spectrometer semi-collection angle of 25 mrad were used for the collection of the EELS signals. The energy resolution of the EELS measurements was 1.2 eV, as estimated from the full-width-half-maximum of the zero-loss peaks. A DualEELS mode was used at a dispersion of 0.1 eV per channel, where the core loss spectra from either Cu or O were calibrated using the zero loss peaks in the low loss spectra. The samples for TEM were prepared by dropcasting aliquots of the disproportionation solution onto lacey carbon grids supplied by EM Resolutions and were left to dry under N_2 blanket.

X-ray photoelectron spectroscopy (XPS)

XPS measurements were performed using a Kratos Axis Ultra DLD spectrometer. The samples were illuminated using X-rays from a monochromated Al Kα source (hν = 1486.7 eV) and detected at a take-off angle of 90°. The resolution, binding energy referencing, and transmission function of the analyser were determined using a clean polycrystalline Ag foil. XPS peak fitting was carried out using the CasaXPS software (Voigt -mixed Gaussian−Lorentzian line shapes and a Shirley background). The peaks were corrected with respect to C 1*s* at 284.7 eV due to the use of the charge neutraliser to avoid surface charging.

Synthetic Procedures

Typical procedure for the self-deoxygenating aqueous Cu-RDRP of NiPAm with targeted DP_n=50.

Conditions*:* [I] : [NiPAm] : [Cu(I)Br] : [Me_6Tren] = 1 : 50 : 0.4 : 0.4.

A vial (***solution A***) was charged with 0.7 g NiPAm (50 eq., 6.186 mmol), 28.8 mg (1 eq., 0.124 mmol) water-soluble initiator (2, 3-dihydroxypropyl 2-bromo-2-methylpropanoate) and **5.3 mL DI-H_2O**. The solution was sonicated until total dissolution of NiPAm. In parallel, a second glass vial (***solution B - disproportionation***) was charged with a stirrer bar, Cu(I)Br (0.4 eq., 0.0495 mmol, 7.1 mg), **1 mL** of DI-H_2O and Me_6Tren (0.4 eq., 0.0495 mmol, 13.3 µL). The vial was septum-sealed, and the disproportionation reaction was left to commence in ice-bath (0-1 ^oC) with **900 rpm stirring** applied for **60 seconds**. Instantly, ***solution B*** became blue (indicating formation of Cu(II)) and black/purple particles (Cu(0) particles) were formed, indicating the successful disproportionation of Cu(I) towards Cu(II) and Cu(0). After 60 seconds, the solution containing the monomer and the initiator (***solution A***) was transferred into the disproportionation solution (***solution B***) through a plastic syringe and the polymerization was left to commence for 12 minutes. It should be noted that while ***solution A*** was being was transferred in ***solution B*** (disproportionation solution), an exit needle was used to facilitate the solution transfer. After 12 minutes, 0.6 mL of the polymerization solution were taken; 0.2 mL were diluted in D_2O for ^1H NMR analysis, while 0.4 mL were diluted in DMF, passed through a neutral alumina column for the removal of copper traces and were filtered for DMF-SEC characterization.

Typical procedure for the self-deoxygenating aqueous Cu-RDRP of NiPAm with targeted DP_n=50 (open-to-air)

Conditions*:* [I] : [NiPAm] : [Cu(I)Br] : [Me_6Tren] = 1 : 50 : 0.4 : 0.4.

A vial (***solution A***) was charged with 0.7 g NiPAm (50 eq., 6.186 mmol), 28.8 mg (1 eq., 0.124 mmol) water-soluble initiator (2, 3-dihydroxypropyl 2-bromo-2-methylpropanoate) and **5.3 mL DI-H_2O**. The solution was sonicated until total dissolution of NiPAm. In parallel, a second

glass vial (***solution B - disproportionation***) was charged with a stirrer bar, Cu(I)Br (0.4 eq., 0.0495 mmol, 7.1 mg), **1 mL** of DI-H_2O and Me_6Tren (0.4 eq., 0.0495 mmol, 13.3 μL) and the disproportionation reaction was left to commence in ice-bath (0-1 ^oC) with **900 rpm stirring** applied for **60 seconds**. Instantly, ***solution B*** became blue (indicating Cu(II)) and black/purple particles (Cu(0) particles) were formed. After 60 seconds, the solution containing the monomer and the initiator (***solution A***) was transferred in the disproportionation solution (***solution B***) through a plastic syringe and the polymerization was left to commence for 12 minutes. After 12 minutes, 0.6 mL of the polymerization solution were taken; 0.2 mL were diluted in D_2O for ^1H NMR analysis, while 0.4 mL were diluted in DMF, passed through a neutral alumina column for the removal of copper traces and were filtered for DMF-SEC characterization.

Typical procedure for the self-deoxygenating aqueous Cu-RDRP of $PEGA_{480}$ with targeted DP_n=20

Conditions*:* [I] : [$PEGA_{480}$] : [Cu(I)Br] : [Me_6Tren] = 1 : 20 : 0.4 : 0.4.

A vial (***solution A***) was charged with 0.7 mL $PEGA_{480}$ (20 eq., 1.59 mmol), 19.2 mg (1 eq., 0.08 mmol) water-soluble initiator (2, 3-dihydroxypropyl 2-bromo-2-methylpropanoate) and **5.3 mL DI-H_2O**. In parallel, a second glass vial (***solution B - disproportionation***) was charged with a stirrer bar, Cu(I)Br (0.4 eq., 0.0318 mmol, 4.6 mg), **1 mL** of DI-H_2O and Me_6Tren (0.4 eq., 0.0318 mmol, 8.5 μL). The vial was septum-sealed, and the disproportionation reaction was left to commence in ice-bath (0-1 ^oC) with **900 rpm stirring** applied for **60 seconds**. Instantly, ***solution B*** becomes blue (indicating Cu(II)) and black/purple particles (Cu(0) particles) are formed, indicating the successful disproportionation of Cu(I) towards Cu(II) and Cu(0). After 60 seconds, the solution containing the monomer and the initiator (***solution A***) was transferred in the disproportionation solution (***solution B***) through a plastic syringe and the polymerization was left to commence for 30 minutes. It should be noted that while ***solution A*** was being was transferred in ***solution B*** (disproportionation solution), an exit needle was used to facilitate the solution transfer. After 30 minutes, 0.6 mL of the polymerization solution were taken; 0.2 mL were diluted in D_2O for ^1H NMR analysis, while 0.4 mL were diluted in DMF, passed through a neutral alumina column for the removal of copper traces and were filtered for DMF-SEC characterization.

Typical procedure for the self-deoxygenating aqueous Cu-RDRP of NAM with targeted DP_n=40

Conditions*:* [I] : [NAM] : [Cu(I)Br] : [Me_6Tren] = 1 : 40 : 0.8 : 0.4.

A vial (***solution A***) was charged with 0.7 mL NAM (40 eq., 5.56 mmol), 33.5 mg (1 eq., 0.14 mmol) water-soluble initiator (2, 3-dihydroxypropyl 2-bromo-2-methylpropanoate) and **5.3 mL DI-H_2O**. In parallel, a second glass vial (***solution B - disproportionation***) was charged with a stirrer bar, Cu(I)Br (0.8 eq., 0.112 mmol, 16 mg), **1 mL** of DI-H_2O and Me_6Tren (0.4 eq., 0.084 mmol, 22.5 μL). The vial was septum-sealed, and the disproportionation reaction was left to commence in ice-bath (0-1 ^{o}C) with **900 rpm stirring** applied for **60 seconds**. Instantly, ***solution B*** becomes blue (indicating formation of Cu(II)) and black/purple particles (Cu(0) particles) formed, indicating the successful disproportionation of Cu(I) towards Cu(II) and Cu(0). After 60 seconds, the solution containing the monomer and the initiator (***solution A***) was transferred in the disproportionation solution (***solution B***) through a plastic syringe and the polymerization was left to commence for 4 hours. It should be noted that while ***solution A*** was being was transferred in ***solution B*** (disproportionation solution), an exit needle was used to facilitate the solution transfer. After 4 hours, 0.6 mL of the polymerization solution were taken; 0.2 mL were diluted in D_2O for ^{1}H NMR analysis, while 0.4 mL were diluted in DMF, passed through a neutral alumina column for the removal of copper traces and were filtered for DMF-SEC characterization.

Typical procedure for the self-deoxygenating aqueous Cu-RDRP of HEAm with targeted DP_n=100

Conditions*:* [I] : [HEAm] : [Cu(I)Br] : [Me_6Tren] = 1 : 100 : 0.8 : 0.4.

A vial (***solution A***) was charged with 0.7 mL HEAm (100 eq., 6.75 mmol), 14.6 mg (1 eq., 0.067 mmol) water-soluble initiator (2, 3-dihydroxypropyl 2-bromo-2-methylpropanoate) and **5.3 mL DI-H_2O**. In parallel, a second glass vial (***solution B - disproportionation***) was charged with a stirrer bar, Cu(I)Br (0.8 eq., 0.054 mmol, 7.75 mg), **1 mL** of DI-H_2O and Me_6Tren (0.4 eq., 0.027 mmol, 7.2 μL). The vial was septum-sealed, and the disproportionation reaction was left to commence in ice-bath (0-1 ^{o}C) with **900 rpm stirring** applied for **60 seconds**. Instantly, ***solution B*** becomes blue (indicating Cu(II)) and black/purple particles (Cu(0) particles) are

formed, indicating the successful disproportionation of Cu(I) towards Cu(II) and Cu(0). After 60 seconds, the solution containing the monomer and the initiator (***solution A***) was transferred in the disproportionation solution (***solution B***) through a plastic syringe and the polymerization was left to commence for 15 minutes. It should be noted that while ***solution A*** was being was transferred in ***solution B*** (disproportionation solution), an exit needle was used to facilitate the solution transfer. After 15 minutes, 0.6 mL of the polymerization solution were taken; 0.2 mL were diluted in D_2O for ^1H NMR analysis, while 0.4 mL were diluted in DMF, passed through a neutral alumina column for the removal of copper traces and were filtered for DMF-SEC characterization.

Typical procedure for the self-deoxygenating aqueous Cu-RDRP of DMA with targeted DP_n=80

Conditions*:* [I] : [DMA] : [Cu(I)Br] : [Me_6Tren] = 1 : 80 : 0.8 : 0.4.

A vial (***solution A***) was charged with 0.7 mL DMA (80 eq., 6.79 mmol), 12.2 mg (1 eq., 0.085 mmol) water-soluble initiator (2, 3-dihydroxypropyl 2-bromo-2-methylpropanoate) and **5.3 mL DI-H_2O**. In parallel, a second glass vial (***solution B - disproportionation***) was charged with a stirrer bar, Cu(I)Br (0.8 eq., 0.068 mmol, 9.75 mg), **1 mL** of DI-H_2O and Me_6Tren (0.4 eq., 0.034 mmol, 9 μL). The vial was septum-sealed, and the disproportionation reaction was left to commence in ice-bath (0-1 ^oC) with **900 rpm stirring** applied for **60 seconds**. Instantly, ***solution B*** becomes blue (indicating Cu(II)) and black/purple particles (Cu(0) particles) are formed, indicating the successful disproportionation of Cu(I) towards Cu(II) and Cu(0). After 60 seconds, the solution containing the monomer and the initiator (***solution A***) was transferred in the disproportionation solution (***solution B***) through a plastic syringe and the polymerization was left to commence for 20 minutes. It should be noted that while ***solution A*** was being was transferred in ***solution B*** (disproportionation solution), an exit needle was used to facilitate the solution transfer. After 20 minutes, 0.6 mL of the polymerization solution were taken; 0.2 mL were diluted in D_2O for ^1H NMR analysis, while 0.4 mL were diluted in DMF, passed through a neutral alumina column for the removal of copper traces and were filtered for DMF-SEC characterization.

Typical procedure for the self-deoxygenating aqueous Cu-RDRP of PHEAm (8-arm) star-shaped polymers with overall targeted DP_n=160

Conditions*:* [I] : [HEAm] : [Cu(I)Br] : [Me_6Tren] = 1 : 160 : 6.4 : 4.2.

The multi-functional (8-arms-see **Scheme S2** below) initiator (64.7 mg, 1 eq., 0.042mmol) was dissolved in 4 mL DMSO and in the same vial, 0.7 mL HEAm (160 eq., 6.75 mmol) which were dissolved in 1.3 mL DI-H_2O were added (***solution A***). In parallel, a second glass vial (***solution B - disproportionation***) was charged with a stirrer bar, Cu(I)Br (6.4 eq., 38.7 mg), **1 mL** of DI-H_2O and Me_6Tren (4.2 eq., 48.5 μL). The vial was septum-sealed, and the disproportionation reaction was left to commence in ice-bath (0-1 ^oC) with **900 rpm stirring** applied for **60 seconds**. Instantly, ***solution B*** becomes blue (indicating Cu(II)) and black/purple particles (Cu(0) particles) are formed, indicating the successful disproportionation of Cu(I) towards Cu(II) and Cu(0). After 60 seconds, the solution containing the monomer and the initiator (***solution A***) was transferred in the disproportionation solution (***solution B***) through a plastic syringe and the polymerization was left to commence for 90 minutes. It should be noted that while ***solution A*** was being was transferred in ***solution B*** (disproportionation solution), an exit needle was used to facilitate the solution transfer. After 90 minutes, 0.6 mL of the polymerization solution were taken; 0.2 mL were diluted in D_2O for ^1H NMR analysis, while 0.4 mL were diluted in DMF, passed through a neutral alumina column for the removal of copper traces and were filtered for DMF-SEC characterization.

Supporting Figures

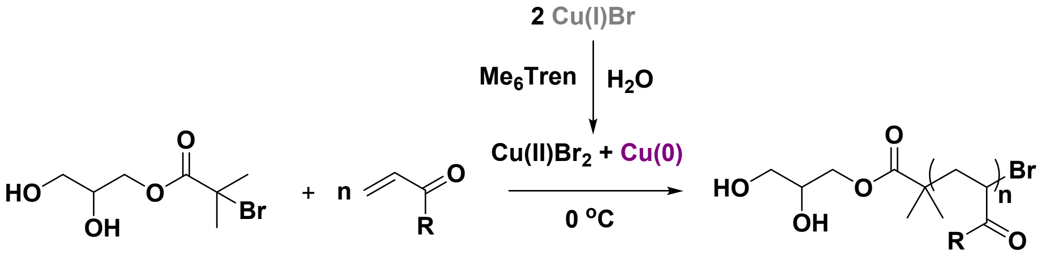

Scheme S1. Reaction scheme of the aqueous Cu-RDRP of acrylamides utilizing the pre-disproportionation of Cu(I)/Me_6Tren in H_2O at 0°C.

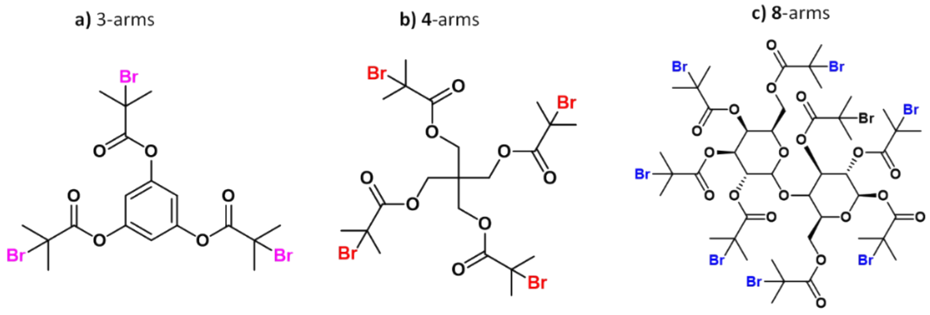

Scheme S2. Chemical structures for the different multi-functional initiators used for the synthesis of star-shaped polymers.

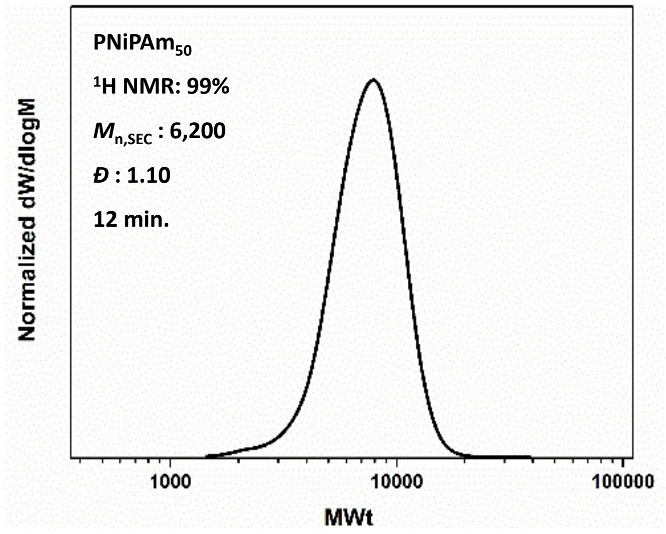

Figure S1. DMF-SEC derived molecular weight distribution of PNiPAm with targeted DP_n = 50 synthesized *via* N_2-deoxygenated aqueous Cu-RDRP with the pre-disproportionation of Cu(I)/Me_6Tren in H_2O at 0 °C.

Table S1. ^{1}H NMR and DMF-SEC analysis of PNiPAm with targeted DPs = 100-400 synthesized *via self-deoxygenating* aqueous Cu-RDRP with the pre-disproportionation of Cu(I)/Me_6Tren in H_2O at 0 °C. [a]

DP	^{1}H NMR (%)	$M_{n,SEC}$ g mol^{-1}	$M_{n,th.}$ g mol^{-1}	*Đ*	Time (minutes)	Monomer (w/v %)	[Cu(I)Br]:[L]:[I] (eq.)
50	>99	7,200	5,900	1.10	12	10	0.4 : 0.4 : 1
100	>99	14,800	11,600	1.08	15	10	0.8 : 0.4 : 1
200	>99	25,100	22,900	1.15	60	10	0.8 : 0.4 : 1
400	92	38,600	41,900	1.18	90	20	0.8 : 0.4 : 1

[a] All the polymerizations were conducted in ice bath (at 0-1 °C) and 900 rpm stirring rate. The disproportionation reaction was left to commence for 60 seconds, thereupon the monomer and initiator solution was added *via* a plastic syringe.

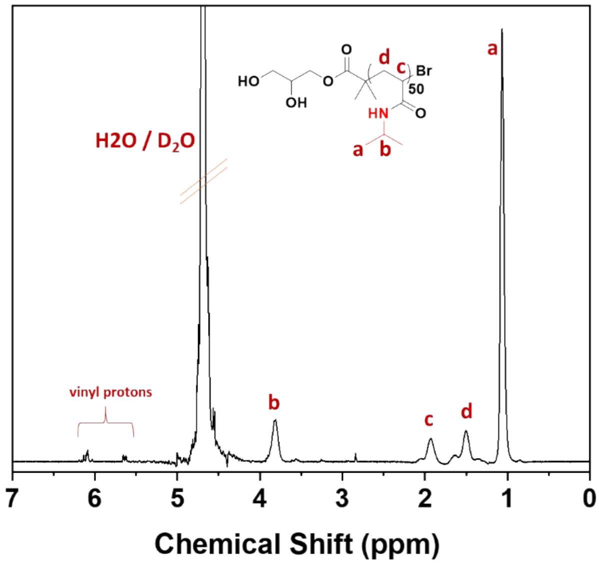

Figure S2. ^{1}H NMR spectrum in D_2O for the PNiPAm with targeted DP_n = 50 synthesized *via self-deoxygenating* aqueous Cu-RDRP with the pre-disproportionation of Cu(I)/Me_6Tren in H_2O at 0 °C. Conversion was determined by comparing the integrals of monomeric vinyl protons (~ 5.5-6.5 ppm) to polymer signal.

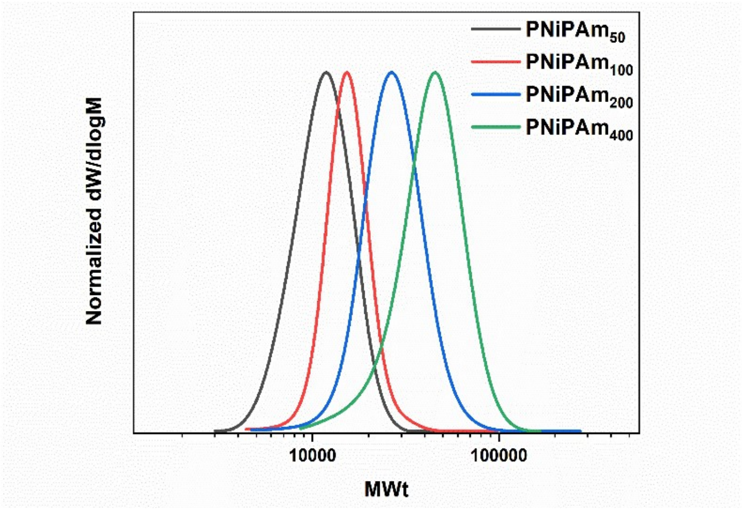

Figure S3a. DMF-SEC derived molecular weight distributions of PNiPAm with targeted DP*s* = 50-400 synthesized *via self-deoxygenated* aqueous Cu-RDRP with the pre-disproportionation of Cu(I)/Me_6Tren in H_2O at 0 ^oC.

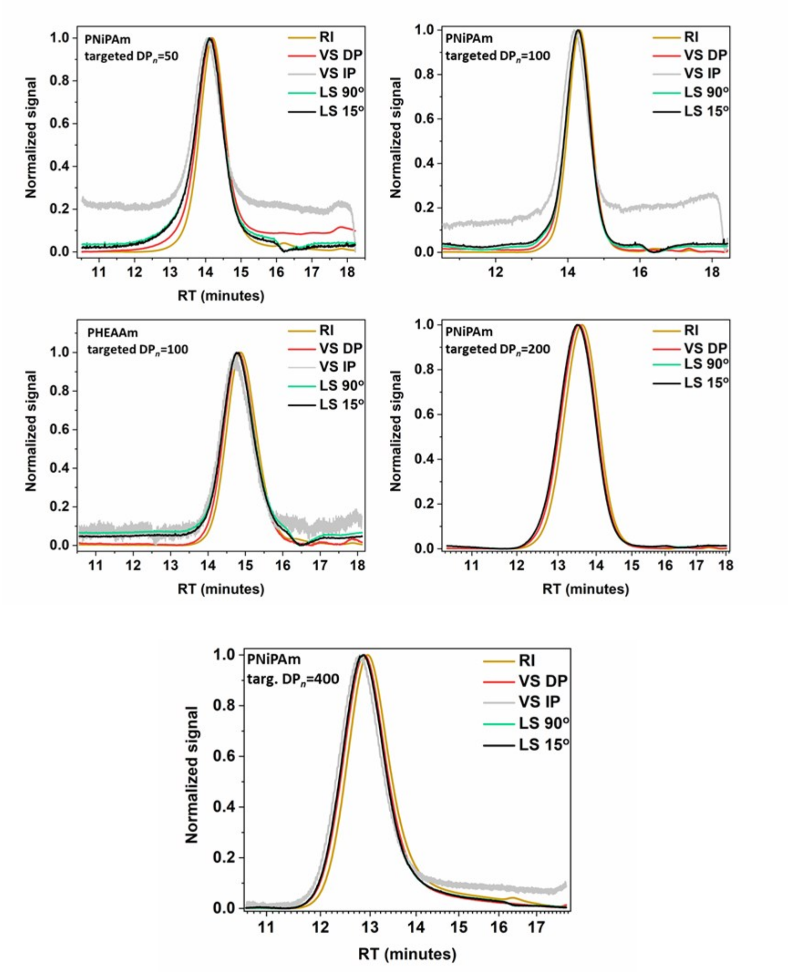

Figure S3b. GPC-SEC derived chromatograms for PNiPAm with different molar masses and PHEAm (targeted DP_n = 100) with light scattering and viscosity data, showing no evidence of background non-controlled polymerization.

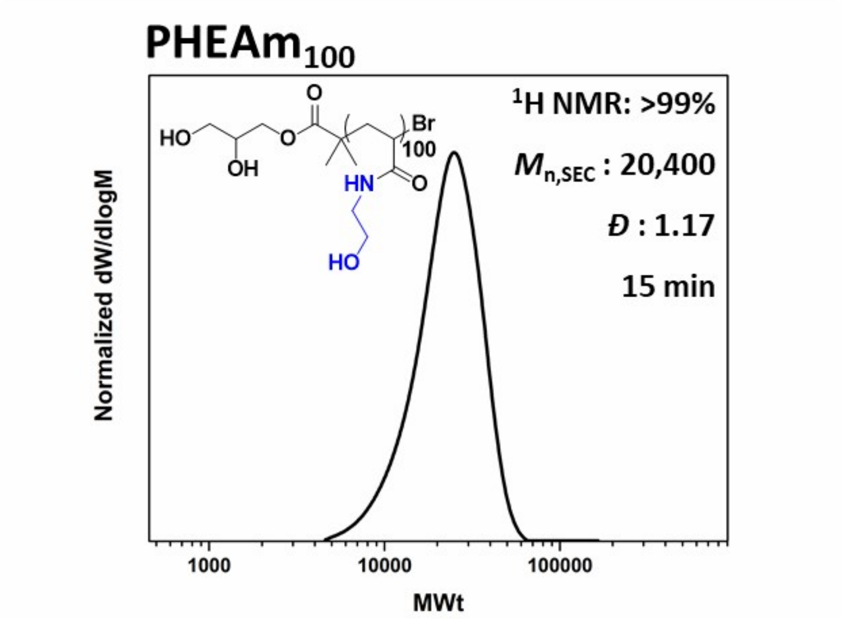

Figure S4a. DMF-SEC derived molecular weight distribution of PHEAm with targeted DP_n = 100 synthesized *via self-deoxygenated* aqueous Cu-RDRP with the pre-disproportionation of Cu(I)/Me_6Tren in H_2O at 0 ^{o}C.

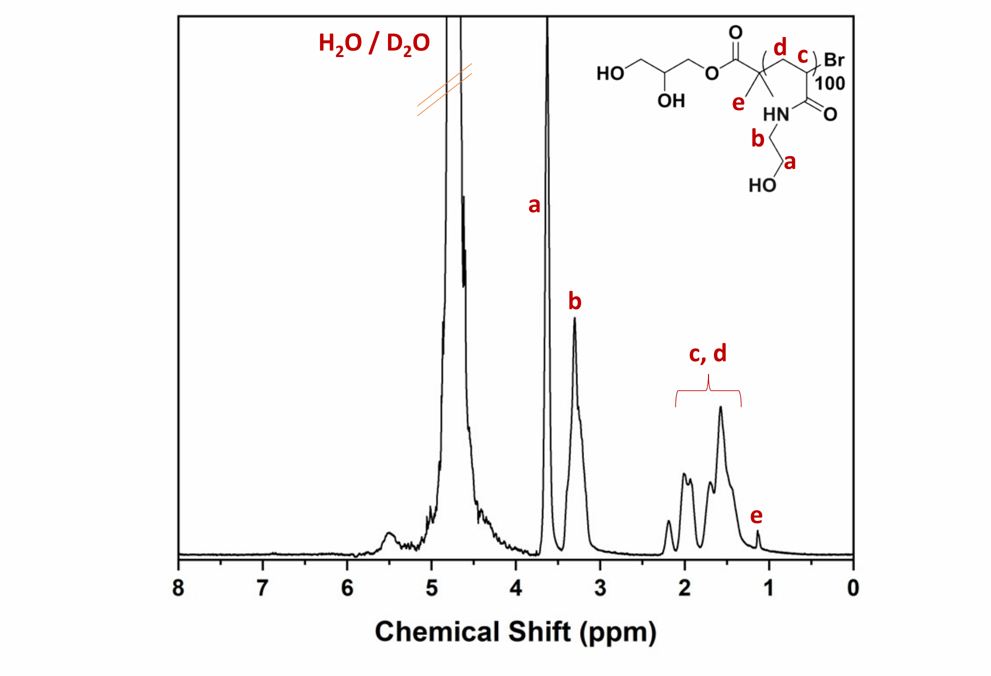

Figure S4b. ^{1}H NMR spectrum in D_2O for the PHEAm with targeted DP_n = 100 synthesized *via self-deoxygenating* aqueous Cu-RDRP with the pre-disproportionation of Cu(I)/Me_6Tren in H_2O at 0 ^{o}C. Conversion was determined by comparing the integrals of monomeric vinyl protons to polymer signal.

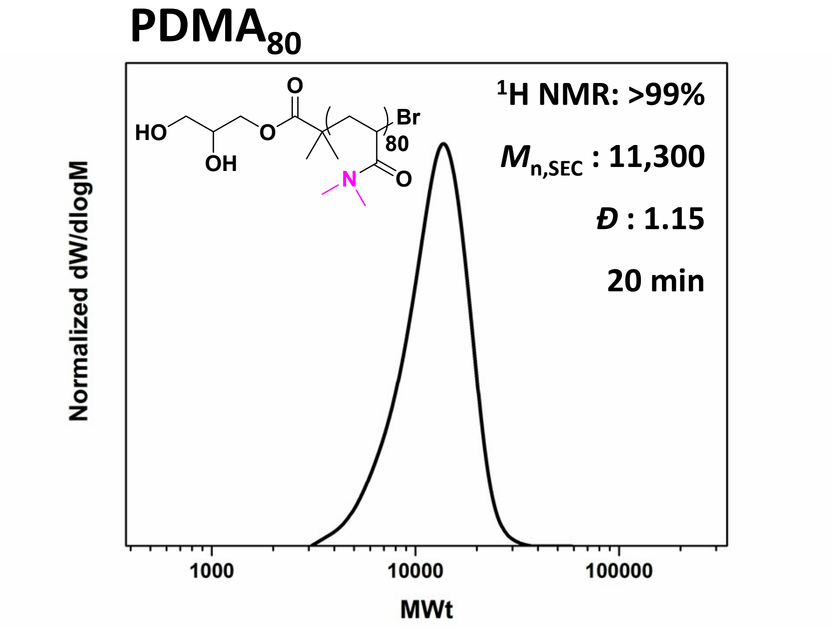

Figure S5. DMF-SEC derived molecular weight distribution of PDMA with targeted DP_n = 80 synthesized *via self-deoxygenated* aqueous Cu-RDRP with the pre-disproportionation of Cu(I)/Me_6Tren in H_2O at 0 ^oC.

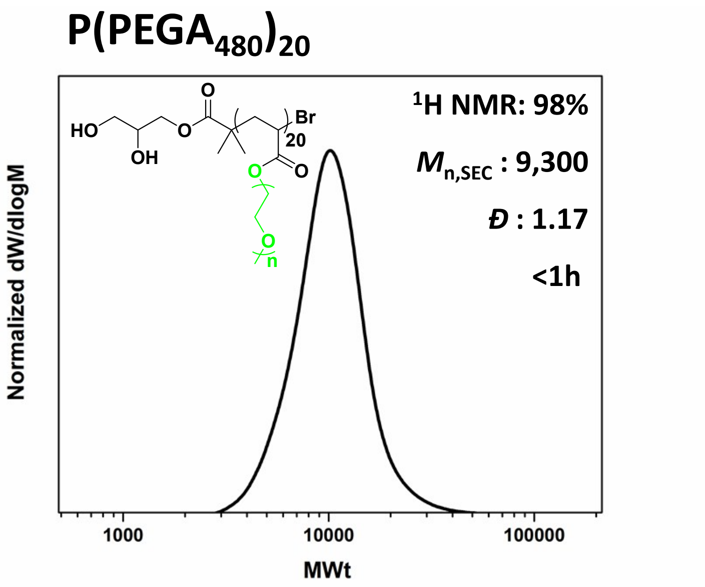

Figure S6a. DMF-SEC derived molecular weight distribution of P($PEGA_{480}$) with targeted DP_n = 20 synthesized *via self-deoxygenated* aqueous Cu-RDRP with the pre-disproportionation of Cu(I)/Me_6Tren in H_2O at 0 ^oC.

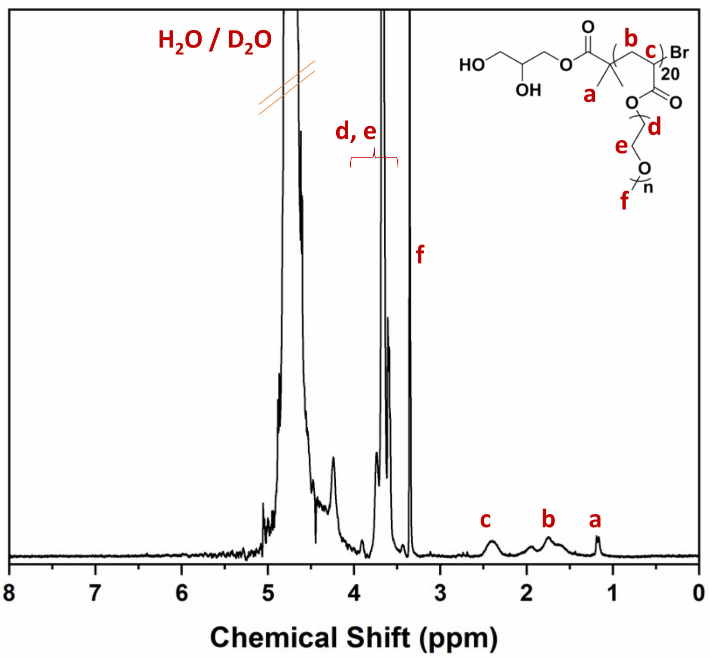

Figure S6b. ^{1}H NMR spectrum in D_2O for the P(PEGA$_{480}$) with targeted DP$_n$ = 20 synthesized *via self-deoxygenating* aqueous Cu-RDRP with the pre-disproportionation of Cu(I)/Me$_6$Tren in H_2O at 0 °C. Conversion was determined by comparing the integrals of monomeric vinyl protons to polymer signal.

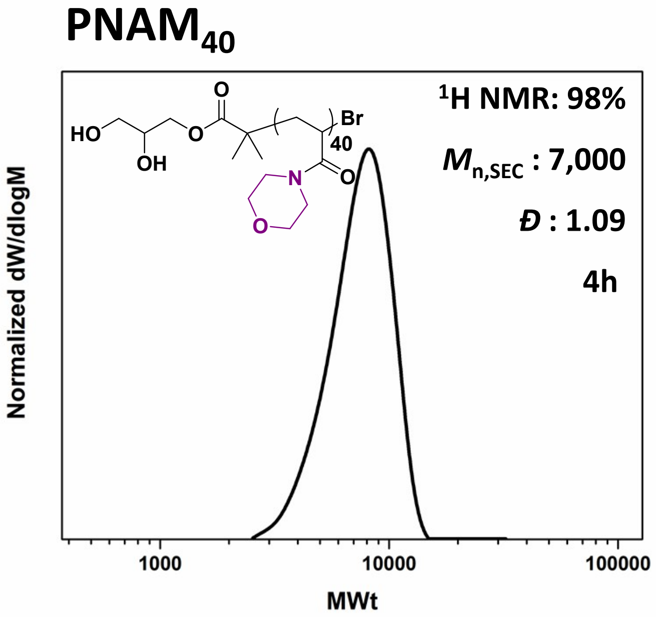

Figure S7a. DMF-SEC derived molecular weight distribution of P(NAM) with targeted DP$_n$ = 40 synthesized *via self-deoxygenated* aqueous Cu-RDRP with the pre-disproportionation of Cu(I)/Me$_6$Tren in H_2O at 0 °C.

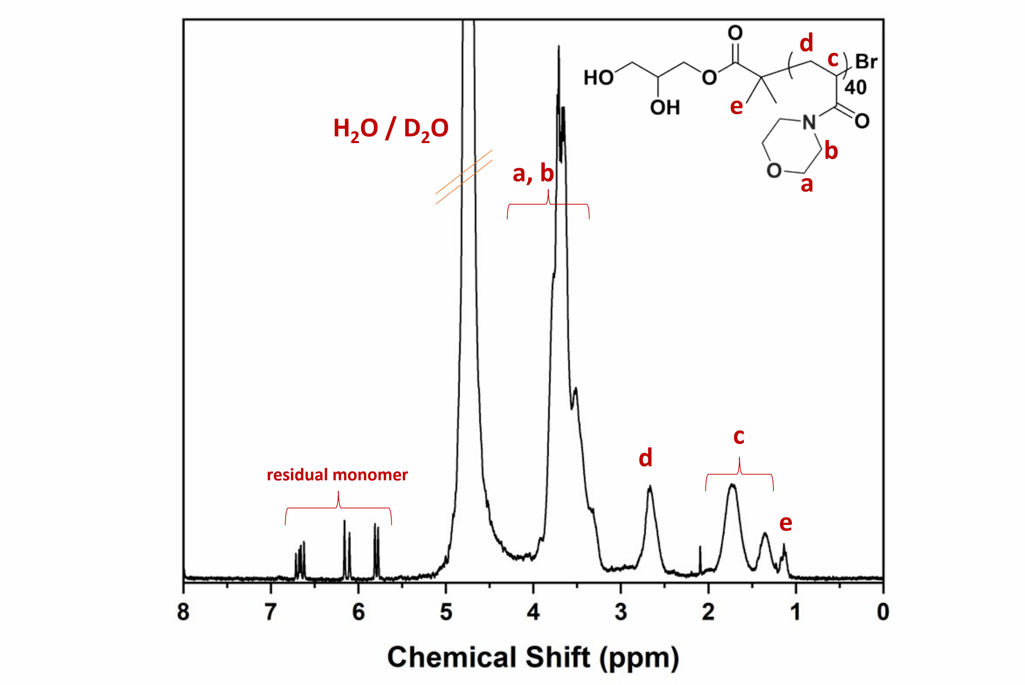

Figure S7b. ^{1}H NMR spectrum in D_2O for the PNAM with targeted DP$_n$ = 40 synthesized *via self-deoxygenating* aqueous Cu-RDRP with the pre-disproportionation of Cu(I)/Me_6Tren in H_2O at 0°C. Conversion was determined by comparing the integrals of monomeric vinyl protons (~5.5 – 6.8 ppm) from the residual monomer to polymer signal.

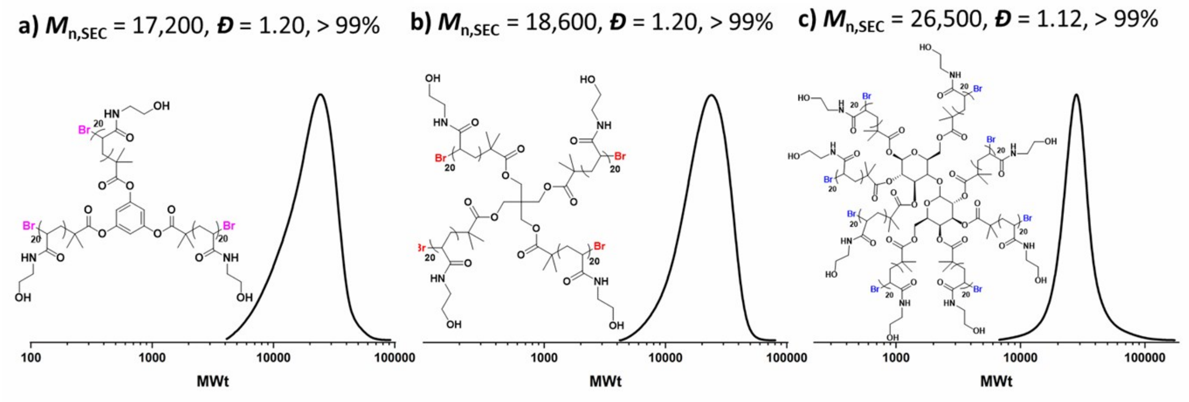

Figure S8. DMF-SEC derived molecular weight distributions for the star-shaped PHEAm utilizing **a**) 3-arm initiator and targeted overall DP$_n$=60, **b**) 4-arm initiator and targeted overall DP$_n$=80 and **c**) 8-arm initiator and targeted overall DP$_n$=160, synthesized *via self-deoxygenating* Cu-RDRP in water – organic solvent mixtures.

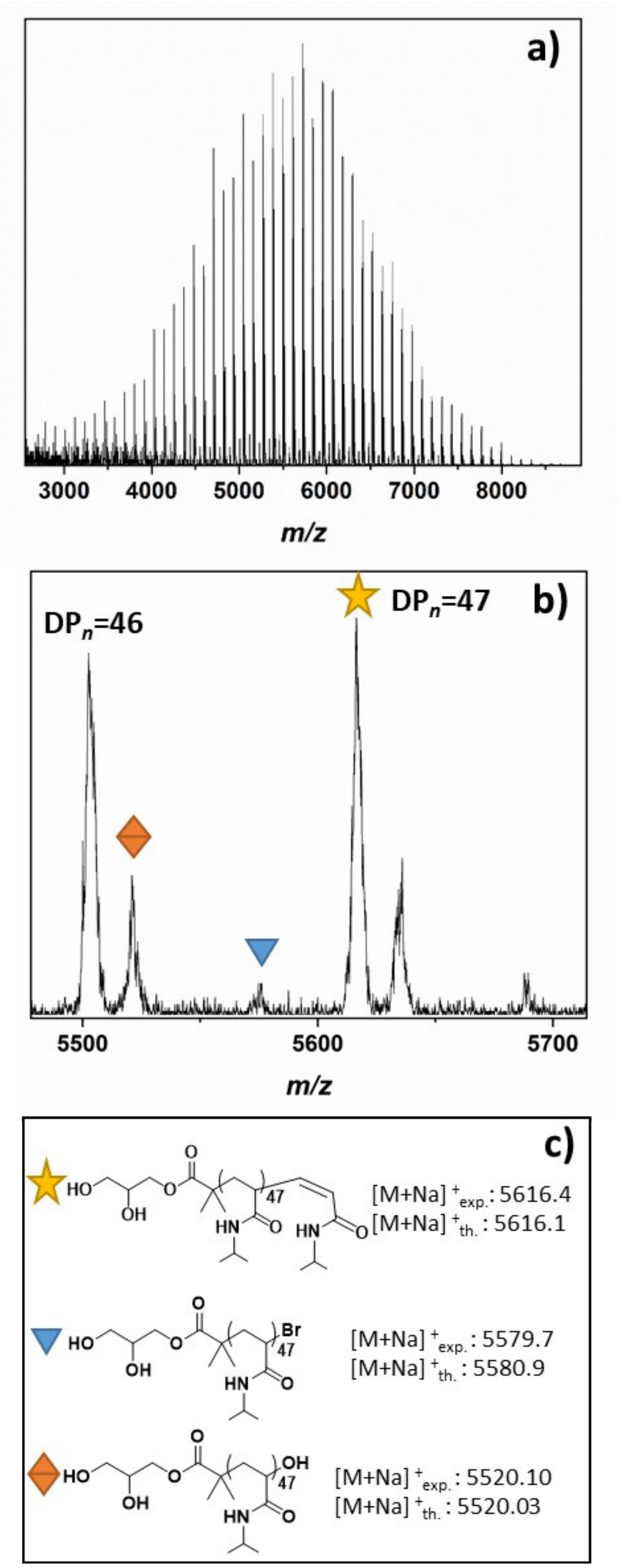

Figure S9a. MALDI-ToF spectrum of PNiPAm with targeted DP$_n$ = 50 and actual DP$_n$ = 47 revealing that he predominant single peak distribution corresponds to Br-eliminated chains ([M+Na]$^+$=5616.4). The presence of –OH terminated chains ([M+Na]$^+$=5520.10) and –Br terminated ([M+Na]$^+$=5579.7) chains is evident.

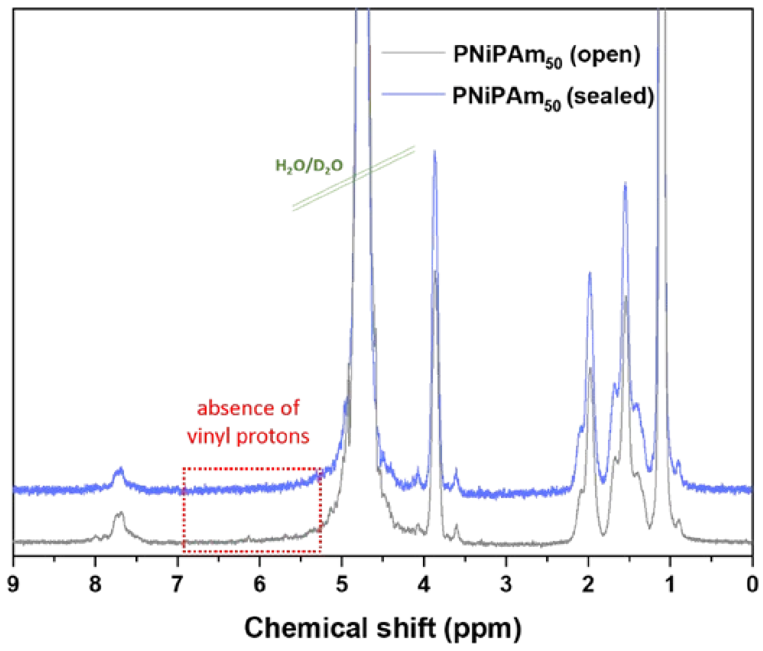

Figure S9b. ^{1}H NMR spectra for PNiPAm (targeted DPn = 50) synthesized in sealed vessel (blue) and open to air (grey) showing the absence of vinyl protons that would indicate elimination.

Table S2. ^{1}H NMR and DMF-SEC analysis of PNiPAm with targeted DP_n = 50 synthesized *via self-deoxygenating* aqueous Cu-RDRP with different concentrations of the disproportionation solution. [a]

**Disproportionation Volume (mL)**	**Conversion (%)** [b]	$M_{n,SEC}$ [c]	$M_{n,th.}$ **g mol**$^{-1}$	*Đ*
1	>99	16,200	11,800	1.08
2	97	16,700	11,800	1.15
3	15	16,900	11,800	1.18
5	0	N/A	N/A	N/A

[a] In all polymerizations the monomer concentration was 10 *w/v* %. [b] Conversion was calculated *via* ^{1}H NMR in D_2O. [c] Determined by DMF-SEC analysis and expressed as molecular weight equivalents to PMMA narrow molecular weight standards.

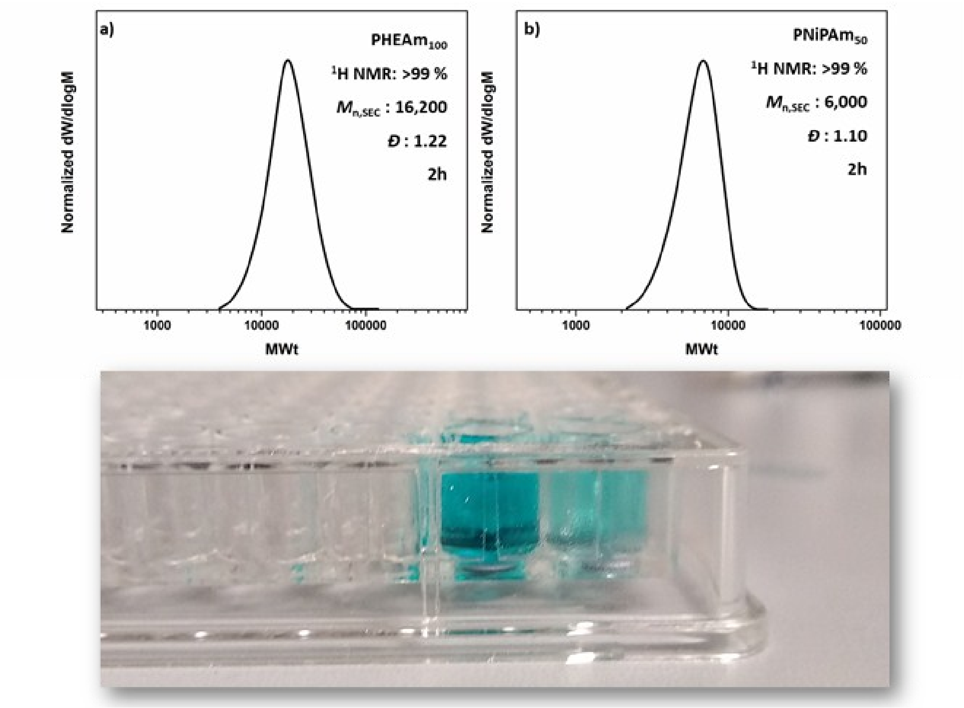

Figure S10. DMF-SEC derived molecular weight distributions for the low-volume **a**) PHEAm with targeted DP$_n$ = 100 and **b**) PNiPAm with targeted DP$_n$ = 50 (top) and reaction setup in 96-well plate (bottom).

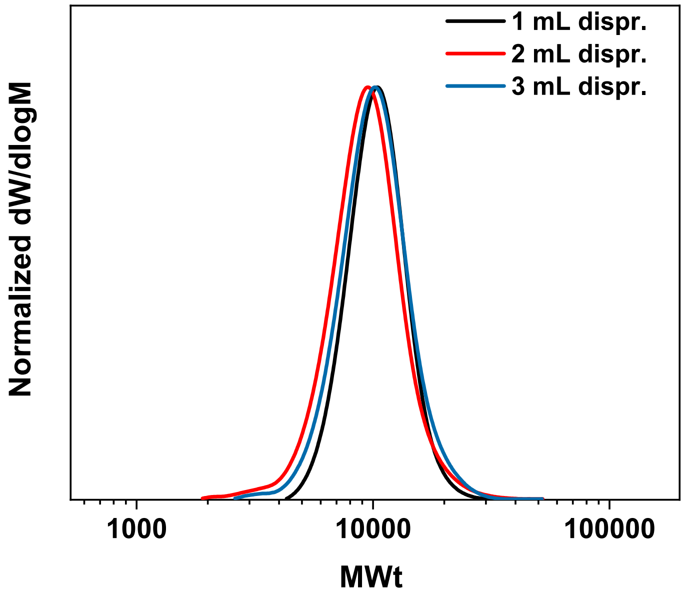

Figure S11. DMF-SEC derived molecular weight distributions of PHEAm with targeted DP$_n$ = 50 synthesized *via self-deoxygenated* aqueous Cu-RDRP with different concentrations of the disproportionation solution.

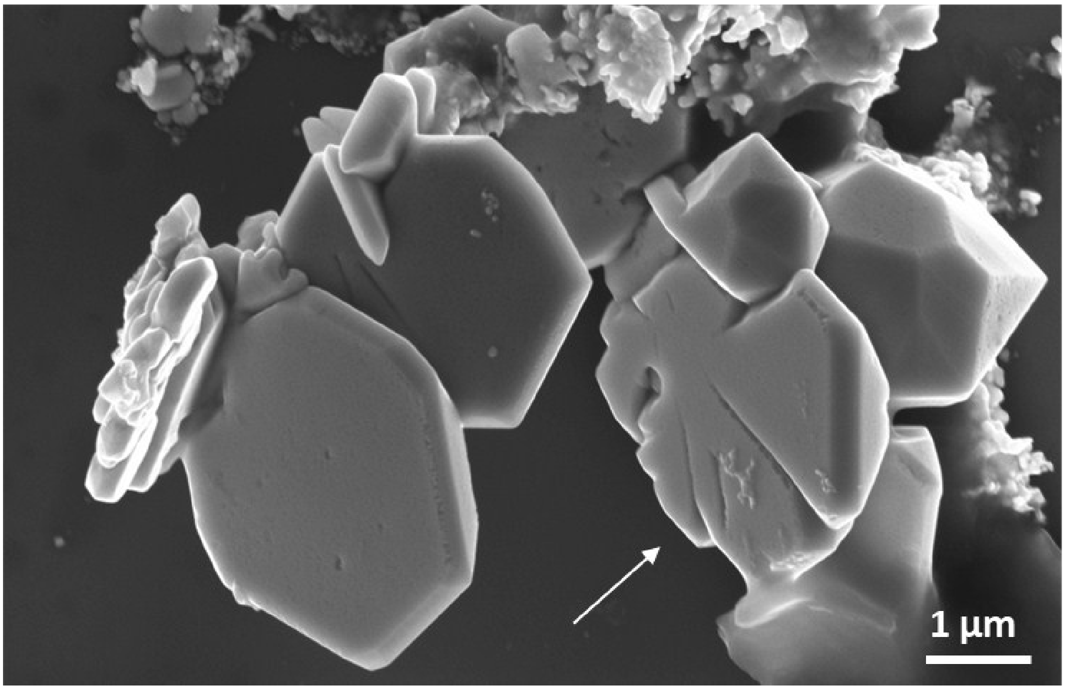

Figure S12. SEM image illustrating the morphological alterations of the faceted crystal-like Cu-particles.

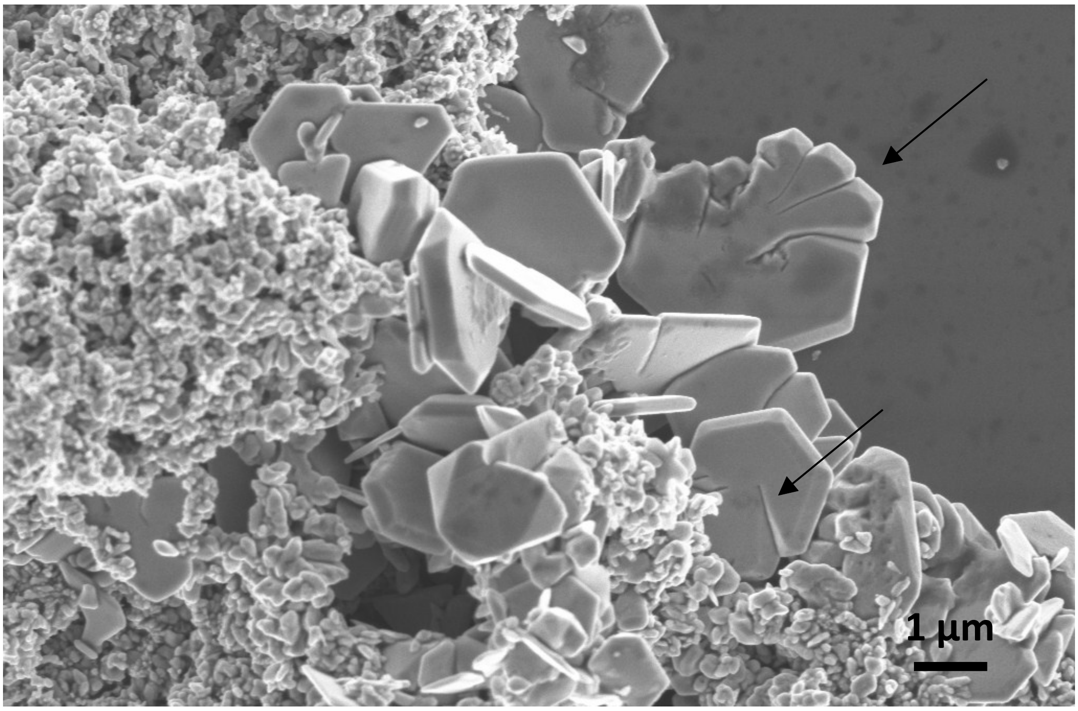

Figure S13. SEM image illustrating the morphological alterations of the faceted crystal-like Cu-particles in different sites.

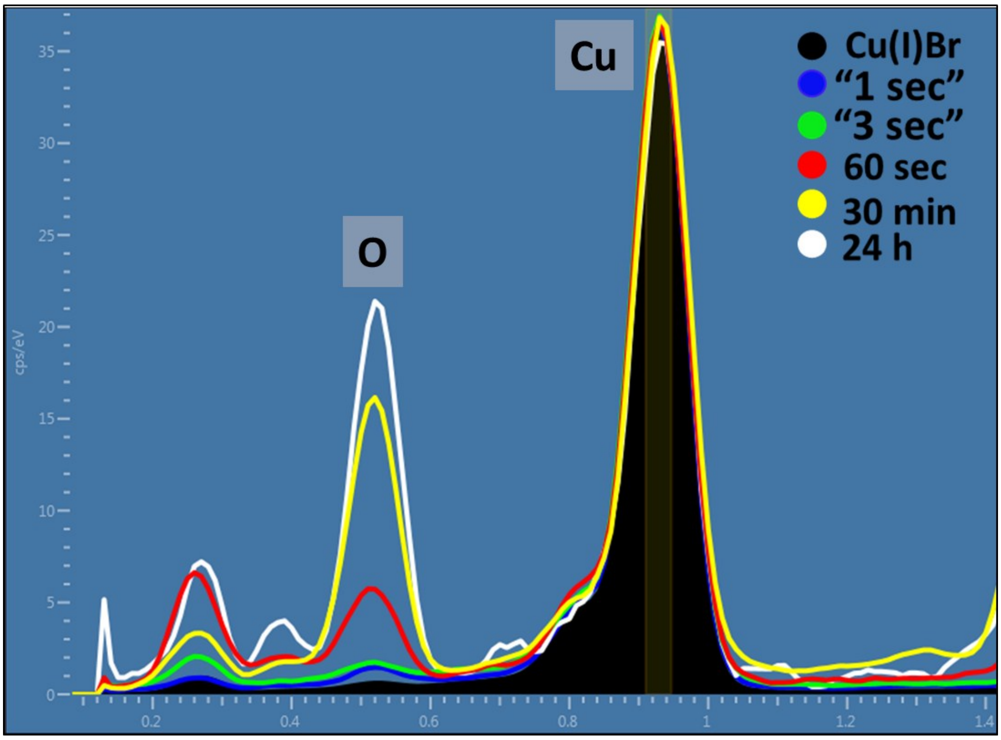

Figure S14. Normalized EDX spectra showing the distinctive copper peak (0.9 keV) and the increase of the oxygen peak (0.5 keV) for Cu(I)Br and the disproportionation precipitate at different times.

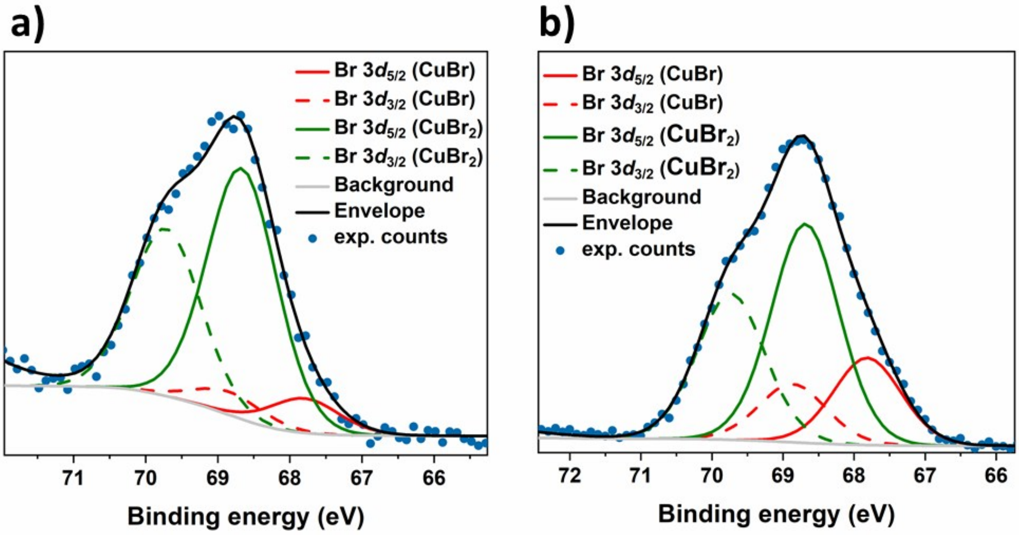

Figure S15. High resolution XPS of the Br 3*d* region revealing the presence of CuBr and $CuBr_2$ in both **a)** the disproportionation precipitate and **b)** supernatant.

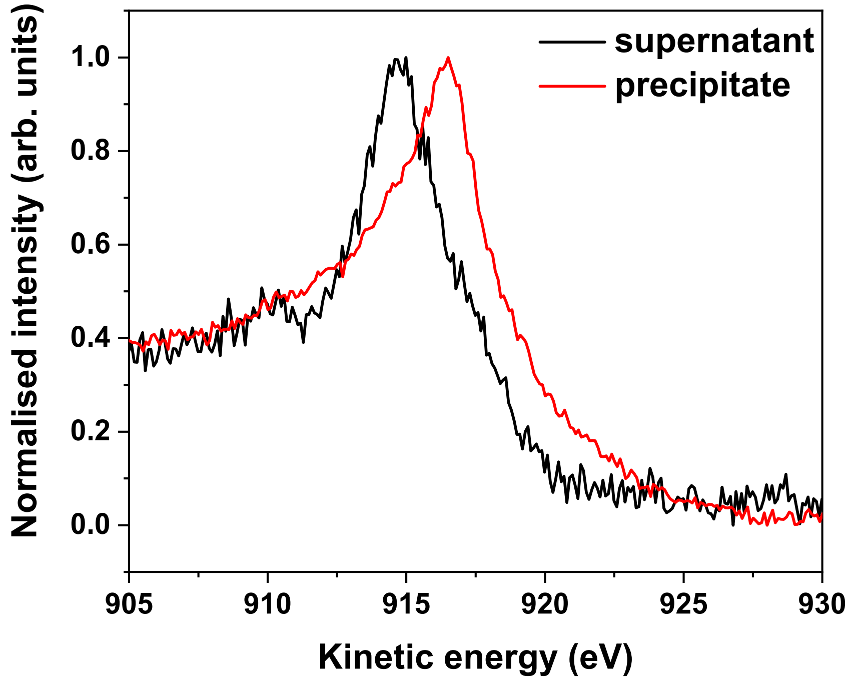

Figure S16. Cu LMM Auger spectra of the disproportionation precipitate and the supernatant after 60 seconds of the reaction. The shift in the position of the maximum intensity reflects a decrease in the relative amount of Cu(II) states ($CuBr_2$, CuO, $Cu(OH)_2$) in the supernatant to a higher proportion of Cu(0) or Cu(I) in the precipitate.[3]

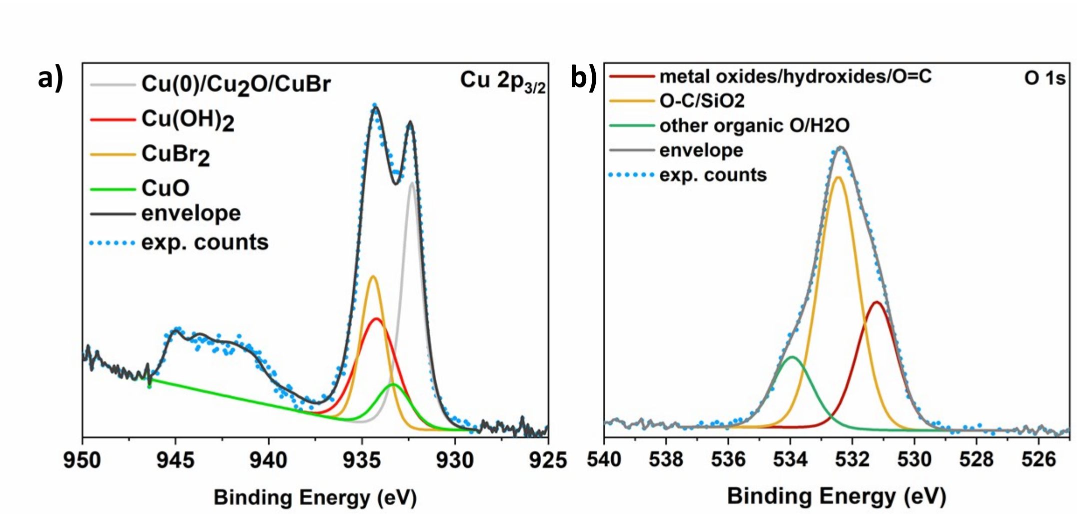

Figure S17. XPS core level **a)** Cu $2p_{3/2}$ and **b)** O 1*s* spectra of the disproportionation supernatant solution after 60 seconds of the reaction. The features between 940 eV and 945 eV are due to shake-up peaks from Cu^{2+} states.[3]

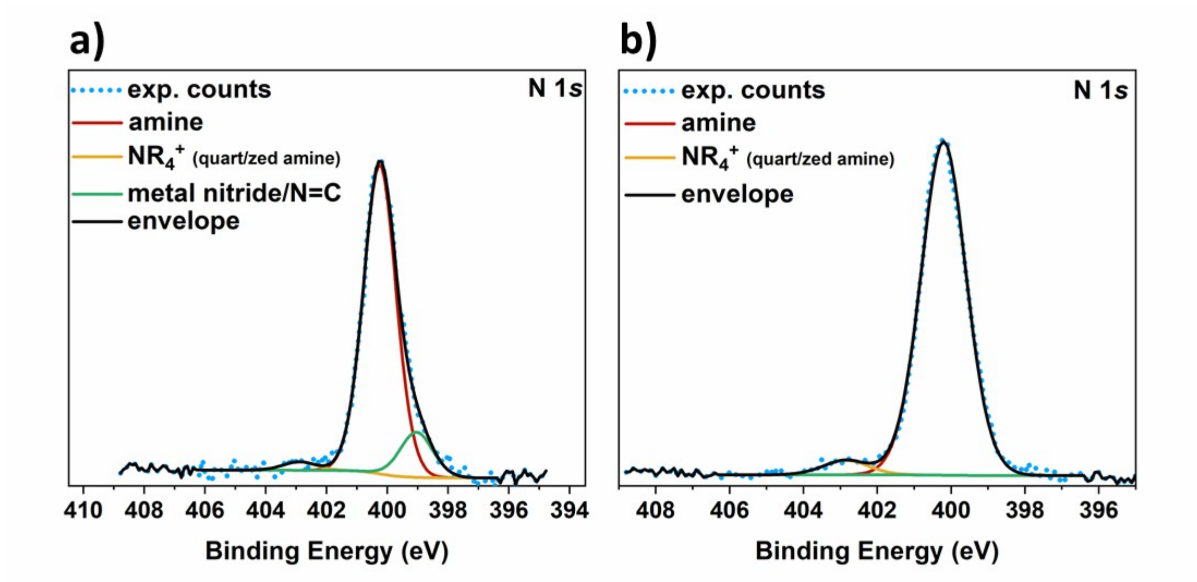

Figure S18. N 1*s* XPS spectra of the disproportionation **a)** precipitate and **b)** supernatant after 60 seconds of the reaction.

Table S3. XPS derived elemental composition for the disproportionation supernatant and precipitate after 60 sec of the reaction in the presence of oxygen.

	Cu	O	N	C	Br	Si [a]
**Supernatant**	3.36	12.85	13.42	64.29	3.62	2.46
**Precipitate**	11.79	23.44	9.42	48.75	1.91	4.69

[a] From silicon wafer

Table S4. XPS derived composition of the disproportionation precipitate after 60 sec of the reaction in the presence of oxygen.

**Precipitate**			
**Cu $2p_{3/2}$**			
**Binding energy (eV)**	**% of region**	**Environment**	**% of Cu $2p_{3/2}$**
932.48	43.35	$Cu(0)/Cu_2O/CuBr$	43.35
934.4	14.38	$Cu(OH)_2$	19.88
939.03	1.44	$Cu(OH)_2$	

941.93	2.92	$Cu(OH)_2$	
943.85	1.14	$Cu(OH)_2$	
934.26	8.09	$CuBr_2$	11.32
941.82	0.55	$CuBr_2$	
943.14	0.96	$CuBr_2$	
945.06	1.72	$CuBr_2$	
933.54	7.8	CuO	25.46
934.91	8.31	CuO	
940.95	0.76	CuO	
942.08	7.07	CuO	
944.13	1.52	CuO	

O 1*s*		
**Binding energy (eV)**	**% of region**	**Environment**
530.72	39.09	Metal oxides/hydroxides/ O=C
531.86	57.93	O-C/SiO_2
533.45	2.99	Other organic O/H_2O

N 1*s*		
**Binding energy (eV)**	**% of region**	**Environment**
400.25	85.22	Amine
402.89	2.34	$NR4^+$ (quarternized amine)
399.04	12.44	Metal nitride or N=C

**C 1*s***		
**Binding energy (eV)**	**% of region**	**Environment**
284.8	18.87	C-C/C-H
285.4	20.49	C-N/C-Br
286.15	48.14	C-OH/C-O-C
287.7	5.74	C=O
289.06	6.76	O=C-O

**Br 3*d***		
**Binding energy (eV)**	**% of region**	**Environment**
67.8	6.16	Br $3d_{5/2}$ - CuBr
68.85	5.96	Br $3d_{3/2}$ - CuBr
68.67	44.69	Br $3d_{5/2}$ - $CuBr_2$
69.72	43.18	Br $3d_{3/2}$ - $CuBr_2$

**Si 2*p***		
**Binding energy (eV)**	**% of region**	**Environment**
98.79	8.66	Si $2p_{3/2}$ - Si(0)
99.4	8.49	Si $2p_{1/2}$ - Si(0)
102.2	41.83	Si $2p_{3/2}$ - SiO_2
102.81	41.03	Si $2p_{1/2}$ - SiO_2

**<u>Supernatant</u>**			
**Cu 2*p*$_{3/2}$**			
**Binding energy (eV)**	**% of region**	**Environment**	**% of Cu 2p$_{3/2}$**
932.31	26.03	Cu(0)/Cu_2O/CuBr	26.03
934.2	19.84	$Cu(OH)_2$	27.44
938.83	1.99	$Cu(OH)_2$	
941.73	4.03	$Cu(OH)_2$	
943.65	1.58	$Cu(OH)_2$	
934.39	17.26	$CuBr_2$	24.15
941.95	1.18	$CuBr_2$	
943.27	2.05	$CuBr_2$	
945.09	3.66	$CuBr_2$	
933.3	6.86	CuO	22.39
934.67	7.31	CuO	
940.71	0.67	CuO	
941.84	6.22	CuO	
943.89	1.33	CuO	

**O 1s**		
**Binding energy (eV)**	**% of region**	**Environment**
531.21	28.43	Metal oxides/hydroxides/ O=C

532.45	55.95	O-C/SiO_2
533.94	15.62	Other organic O/H_2O

**N 1*s***		
**Binding energy (eV)**	**% of region**	**Environment**
400.2	95.78	Amine
402.85	4.22	$NR4^+$ (quarternized amine)

**C 1*s***		
**Binding energy (eV)**	**% of region**	**Environment**
284.8	7.17	C-C/C-H
285.4	19.85	C-N/C-Br
286.15	64.2	C-OH/C-O-C
287.6	4.99	C=O
289.06	3.79	O=C-O

**Br 3*d***		
**Binding energy (eV)**	**% of region**	**Environment**
67.81	14.46	Br $3d_{5/2}$ - CuBr
68.86	13.98	Br $3d_{3/2}$ - CuBr
68.68	36.38	Br $3d_{5/2}$ - $CuBr_2$
69.73	35.18	Br $3d_{3/2}$ - $CuBr_2$

Si 2*p*		
**Binding energy (eV)**	**% of region**	**Environment**
98.97	8.75	Si $2p_{3/2}$ - Si(0)
99.58	8.58	Si $2p_{1/2}$ - Si(0)
102.33	41.74	Si $2p_{3/2}$ - SiO_2
102.94	40.94	Si $2p_{1/2}$ - SiO_2

References:

1 M. Ciampolini and N. Nardi, *Inorg. Chem.*, 1966, **5**, 41–44.

2 S. Perrier, S. P. Armes, X. S. Wang, F. Malet and D. M. Haddleton, *J. Polym. Sci. Part A Polym. Chem.*, 2001, **39**, 1696–1707.

3 A. J. Limer, A. K. Rullay, V. S. Miguel, C. Peinado, S. Keely, E. Fitzpatrick, S. D. Carrington, D. Brayden and D. M. Haddleton, *React. Funct. Polym.*, 2006, **66**, 51–64.

4 M. C. Jones, M. Ranger and J. C. Leroux, *Bioconjug. Chem.*, 2003, **14**, 774–781.
